# Small extracellular vesicles enhance the survival of Sca-1+ cardiac stem cells against ROS-induced ischemic-reoxygenation injury in vitro

**DOI:** 10.1186/s40659-025-00593-7

**Published:** 2025-03-05

**Authors:** Radwa A. Mehanna, Hagar Elkafrawy, Marwa M. Essawy, Samar S. Ibrahim, Ashraf K. Awaad, Nehal A. Khalil, Marwa A. Kholief, Abeer Sallam, Heba A. Hamed, Mona A. Barkat, Mohamed F. ElKady, Eman H. Thabet

**Affiliations:** 1 Medical Physiology Department, Faculty of Medicine, Alexandria University, Alexandria, 21500 Egypt; 2 Center of Excellence for Research in Regenerative Medicine and Applications (CERRMA), Faculty of Medicine, Alexandria University, Alexandria, 21500 Egypt; 3 Medical Biochemistry Department, Faculty of Medicine, Alexandria University, Alexandria, 21500 Egypt; 4 Oral Pathology Department, Faculty of Dentistry, Alexandria University, Alexandria, 21500 Egypt; 5 Biotechnology Department, Center of Excellence for Research in Regenerative Medicine and Applications (CERRMA), Faculty of Medicine, Alexandria University, Alexandria, 21500 Egypt; 6 Molecular Biology Department, Center of Excellence for Research in Regenerative Medicine and Applications (CERRMA), Faculty of Medicine, Alexandria University, Alexandria, 21500 Egypt; 7 Forensic Medicine and Clinical Toxicology Department, Faculty of Medicine, Alexandria University, Alexandria, 21500 Egypt; 8 Histology and Cell Biology Department, Faculty of Medicine, Alexandria University, Alexandria, 21500 Egypt; 9 Human Anatomy and Embryology Department, Faculty of Medicine, Alexandria University, Alexandria, 21500 Egypt; 10 Medical Biophysics Department, Faculty of Medicine, Alexandria University, Alexandria, 21500 Egypt

**Keywords:** BMMSCs-derived small extracellular vesicles, Cardiac stem cells, Ischemia-reperfusion injury, miRNA-21-5p, PTEN/pAkt/HIF-1α pathway

## Abstract

**Background:**

Ischemic reperfusion (IR) generates reactive oxygen species (ROS) that inevitably result in myocardial cell death and heart failure. The regenerative power of cardiac progenitor/stem pools (CSCs), especially the Sca1^+^ population, in response to IR injury remains unclear.

**Methods:**

Our work sought to investigate whether small extracellular vesicles (sEVs) isolated from bone marrow-mesenchymal stem cells (BMMSCs) could rescue CSCs, specifically Sca-1+/CSCs, from IR by increasing their proliferative capacity and limiting their apoptosis in vitro. The Sca-1+/CSCs-IR model was induced by the oxygen-glucose deprivation/reoxygenation method (OGD/R). The effects of treatment with BMMSCs-derived sEVs on oxidative stress, cell proliferation, apoptosis, and cell cycle were assessed. To further test the mechanistic action, we assessed the PTEN/pAkt/HIF-1α pathway.

**Results:**

Compared to hypoxic untreated CSCs, BMMSCs-derived sEVs-treated cells had shifted from their quiescent to proliferative phase (*p* > 0.05) and showed decreased apoptosis (*p* < 0.001). sEVs-treated CSCs were predominately in the S phase (11.8 ± 0.9%) (*p* < 0.01). We identified an abundance of miRNA-21-5P in BMMSCs. HIF-1α expression was highest in CSCs treated with sEVs (*p* < 0.05). Moreover, miRNA-21-5p-rich sEVs shifted the redox state, reducing oxidative stress and promoting balance (*p* > 0.05).

**Conclusion:**

Conditioning Sca-1+/CSCs, an essential population in the postnatal heart, with sEVs rich in miRNA-21 robustly enhanced the proliferation, and synthesis phase of the cell cycle, and stabilized HIF-1α while alleviating oxidative stress and apoptosis. Such sEVs rich in miRNA-21-5p can be further used as a preconditioning tool to enhance endogenous Sca-1+/CSCs regeneration in response to IR injury.

**Supplementary Information:**

The online version contains supplementary material available at 10.1186/s40659-025-00593-7.

## Introduction

Myocardial ischemia/reperfusion injury (MIRI) is a clinicopathological criterion defined as insufficiency of coronary blood supply to the heart and subsequent perfusion recovery with reoxygenation. Reperfusion is the mainstay for the survival of myocardial ischemia tissues. However, reperfusion itself induces additional tissue injury and infarct extension [1]. MIRI inevitably leads to ischemic heart disease (IHD), which is currently one of the leading causes of death worldwide. Globally, > 15 million IHD cases are reported each year [2]. On the cellular level, MIRI induces apoptosis and necrosis of cardiomyocytes [3]. 

The regenerative capacity of the post-ischemic heart is contingent on a population of cardiac stem cells (CSCs) activated by hypoxic injury. Unlike the other heart cells, CSCs are undifferentiated, different in their anatomical, functional, and histological characteristics, and under certain circumstances, they are compelled to differentiate into cardiomyocytes [4]. CSCs can principally differentiate into mature cardiomyocytes, vascular lineages, and smooth muscle cells. These cells exist in a multicellular heterogeneous niche embedded in a specialized stroma. CSCs within this niche maintain their self-renewal capacity and are responsive to external stimuli and intercellular communicators. CSCs remain quiescent and factors that activate it are not understood and are debatable in the adult heart [5]. Upon isolation and culture in vitro, CSCs could be expanded and transplanted into damaged myocardial tissue to stimulate new cardiomyocyte formation and rejuvenate the microcirculation of the ischemic myocardium. However, CSCs are often subjected to accelerated apoptosis and oxidative stress when exposed to a hypoxic environment, compromising their survival and hence their transplantation efficacy [6]. Measures to enhance the survival of CSCs and limit their death are currently uninvestigated.

In-vivo, previous studies have proved that bone marrow mesenchymal stem cells (BMMSCs) could reduce intestinal ischemia/reperfusion (I/R) in rats. They also can enhance cardiac functions in IHD animal models and patients [7]. However, BMMSCs have a low survival rate in the host after transplantation, and only a small proportion of transplanted BMMSCs can be detected in the target tissue [8]. Despite these limitations, BMMSCs transplantation is still conspicuous in tissue regeneration due to their paracrine effects. BMMSCs impose their regenerative actions mainly through paracrine actions and secreting extracellular vesicles (EVs) that include small extracellular vesicles (sEVs). sEVs range 30 to 150 nm in diameter and contain functional proteins and miRNAs [9]. EVs promote intercellular communication and fuse with the recipient cells, leading to the altering of their function through delivering proteins, RNAs, miRNAs, and other molecular constituents [10]. They vary in their cargo according to the cell of origin. For instance, those secreted from stem cells tend to carry molecules that potentiate regeneration and cell survival. Several studies have demonstrated that MSC-secreted sEVs have a regenerative effect on injured tissues, including post-myocardial infarction and MIRI [11], due to their anti-apoptotic, anti-fibrotic, and pro-angiogenic roles. However, further validation of these observations is required, as the underlying mechanisms of their specific effects on CSCs remain undetermined. We sought to investigate if BMMCS-sEVs can rescue CSCs in vitro when exposed to hypoxia followed by reoxygenation.

MiRNAs are among the cargo molecules carried by the EVs that play pivotal roles in improving the undesirable consequences of acute myocardial infarction [12]. MiRNAs are endogenous single-stranded non-coding RNAs consisting of 20 ± 2 nucleotides that play critical roles in mRNA silencing and degradation. miRNAs released in MSC sEVs may also regulate the proliferation, differentiation, and survival of CSCs [13]. miRNA-21-5p modulates the function of MSCs by controlling the phosphatase and tensin homolog (PTEN) gene that further regulates cell apoptosis and survival. miRNA-21 plays significant roles in cardiac cell development and death, vascular smooth muscle cell proliferation and apoptosis, and cardiac fibroblast functions [14]. In experimental studies, miRNA-21 protected against ischemia-induced cardiac cell death and IR-induced heart damage [15]. Among the most extensively studied targets of miRNA-21-5p is the PTEN/pAkt pathway, known for its role in suppressing PTEN expression and reducing apoptosis [16]. 

Hypoxia-inducible factor-1α (HIF-1α) is a transcription factor consisting of a constitutively expressed β-subunit and an oxygen-regulated α-subunit. The α-subunit is an oxygen-dependent degradation domain that is degraded by proline-hydroxylase-2 (PHD-2), rendering the α-subunit vulnerable to proteasomal degradation under normoxic cellular conditions, however, stabilized under hypoxic conditions. Once HIF-1α is stabilized the hypoxic responsive element (HRE) [13] acts to control the expressions of genes involved in angiogenesis, glucose metabolism, cell death, and survival, hence maintaining homeostasis under hypoxic conditions [16]. A positive feedback loop between miRNA-21 and HIF-1 activity has been reported in human cardiomyocytes, where hypoxia triggers the expression of miRNA-21 and miRNA-21 in turn, promotes HIF-1α expression and regulates the PTEN/Akt pathway [17]. 

We hypothesize that inducing HIF-1α expression or stabilizing it in CSCs using BMMSC-EVs would further potentiate their survival and regenerative capacity. There are several ways to achieve this effect, including CSCs transfection with HIF-1α or coculturing with EVs derived from stem cells. In this study, we sought to investigate the in-vitro behavior of CSCs under different experimental conditions including hypoxia, reoxygenation, and supplementation with BMMSCs-derived EVs (rich in miRNA-21-5p) upon reoxygenation. To evaluate the effects and efficacy of these conditions treatments we assessed the proliferation capacity, cell cycle, and apoptosis. To further provide a mechanistic analysis, we evaluated PTEN/pAkt/HIF-1α, a survival pathway for most cells and a common target for miRNA-21-5P, under all CSCs’ experimental conditions.

## Materials and methods

### Study design and experimental animals

**Fig. 1:**
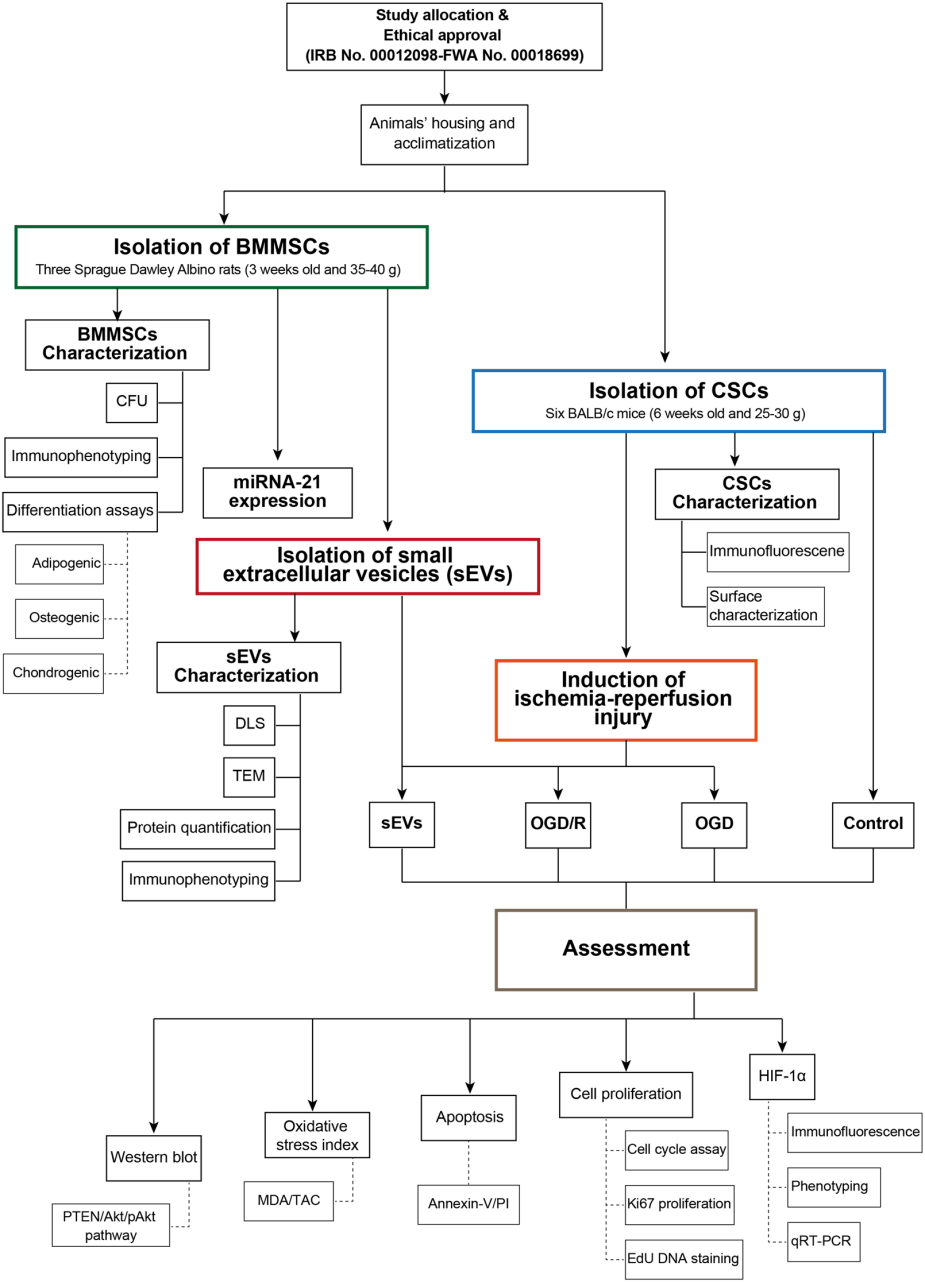
Flow chart of the study design and consecutive steps conducted in the study. BMMSCs: Bone marrow mesenchymal stem cells; CFU: Colony forming unit; CSCs: Cardiac stem cells; DLS: Dynamic light scattering; sEVs: sEVs-treated group; HIF-1α: Hypoxia-inducible factor-1α; MDA: Malondialdehyde; OGD: Oxygen glucose deprivation; OGD/R: Oxygen glucose deprivation/reoxygenation; PI: Propidium iodide; TAC: Total antioxidant capacity; TEM: Transmission electron microscope

The study design with the sequential steps is illustrated in the flow chart (Fig. 1). The present study was conducted at the Center of Excellence for Research in Regenerative Medicine and its Applications (CERRMA) at the Alexandria Faculty of Medicine (AFM). Sprague Dawley Albino rats and BALB/c mice were used for BMMSCs and CSCs isolation, respectively. Animals were purchased and housed at the Experimental Animal House of the Physiology Department, AFM, with food/water ad libitum. All methods were approved by the Alexandria Faculty of Medicine Ethics Committee (Ethics approval number: IRB code 00012098-FWA: No. 00018699; Serial No 0305747, (15/09/2022) and carried out in accordance with the NIH Guide for the Care and Use of Laboratory Animals.

## Isolation of BMMSCs

BMMSCs were isolated using a previously established protocol [18]. Briefly, three Sprague Dawley Albino rats were euthanized, and the bone marrow of the femurs and tibias were flushed using complete culture medium (CCM) supplemented with 10%fetal bovine serum,1% L-glutamine, and 1% penicillin/streptomycin. The flushed cells were cultured in a T25 flask and incubated in a 5% CO_2_ incubator. After 48 h, adherent cells were washed, and the CCM was changed every 2–3 days. (supplementary materials, Sect. 1)

## Characterization of BMMSC

### Colony-forming unit assay

At passage 3, the colony-forming unit (CFU) potential of the BMMSCs was tested [19]. After 14 days of incubation, the CFU was calculated using the following formula: plating efficiency = number of colonies formed/number of plated cells. The optimal CFU for BMMSC culture was over 40%. (supplementary materials, Sect. 2.1)

### Differentiation assays

To assess the multipotency of BMMSCs, in vitro differentiation into osteogenic, chondrogenic, and adipogenic lineages was performed. *(*supplementary material: Sect. 2.2)

#### In vitro osteogenic differentiation

Cells were grown to 80% confluence and incubated in an osteogenic induction medium for 21 days. After induction, cells were fixed in 4% PFA and stained with Alizarin Red Stain for imaging [20]. 

#### In vitro chondrogenic differentiation

BMMSCs were resuspended in chondrogenic induction media, centrifuged, and incubated for 28 days. Media was changed every three days until the chondrogenic pellet was fully differentiated and ready for assessment by Alcian blue stain [21]. The chondrogenic pellet was harvested, embedded in paraffin blocks, sectioned into 5 μm tissue sections on glass slides, and stained for cartilage-specific proteoglycan assessment using Alcian blue 8GX stain.

#### In vitro adipogenic differentiation

BMMSC cells were cultured in CCM and then switched to an adipogenic induction medium for two weeks. The media was then replaced with a differentiation medium containing insulin. After three weeks, cells were fixed and stained by Oil red O for lipid accumulation and visualized by phase contrast microscopy [22]. 

### Immunophenotyping of BMMSCs

BMMSCs were characterized using fluorescent-labeled primary monoclonal antibodies and unconjugated primary antibodies. Cells were trypsinized, washed, and incubated with the following primary antibodies: anti-CD73, CD11b, CD90, CD73, and CD105. Following incubation, the primary unconjugated antibodies were washed, and the corresponding conjugated secondary antibodies were incubated with the stained cells for 45 min in the dark. All stained cells were then washed and resuspended in FACS buffer, and the fluorescence was analyzed using a BD, FACS caliber flow cytometer operated with Cell Quest software (Becton Dickinson, USA) [23]. (supplementary materials, Sect. 2.3).

## Isolation of small extracellular vesicles (sEVs)

sEVs were isolated using a differential ultracentrifugation protocol [24]. Briefly, the BMMSCs CCM was replaced with serum-free media for 48 h, then collected for sEVs isolation. The conditioned media was spun, centrifuged, filtered, and ultracentrifuged. The pellet was resuspended in PBS and stored at -80 °C for further analysis. (supplementary materials, Sect. 3)

## Characterization of small extracellular vesicles (sEVs)

### Dynamic light scattering

A diluted sample of sEVs was sonicated to prevent aggregate formations, then placed in the Zeta-sizer sampling tube for size measurement using Malvern Panalytical Software, UK. The measurements were conducted in triplicate under constant equipment settings [25]. 

### Transmission electron microscopy

The sEVs pellet was dissolved in PBS, loaded to copper grids, and stained with 1% (w/v) phosphotungstic acid (PTA) and examined by transmission electron microscopy (TEM) with an accelerating voltage of 120 kV (JEM-1400 series 120 kV Transmission Electron Microscope, USA) [26]. 

### Protein quantification of small extracellular vesicles (sEVs)

The concentration of sEV proteins was determined using the bicinchoninic acid assay (BCA) kit (Sigma-Aldrich, USA) [27]. 

### Immunophenotyping of small extracellular vesicles (sEVs)

The surface tetraspanin proteins of isolated sEVs were analyzed using magnetic beads from an EVs Isolation Kit Pan (Miltenyi 130-117-039). The beads were incubated with the sEVs, washed, blocked, and stained with anti-CD9, anti-CD63, and anti-CD81 antibodies. MitoTracker probes were used as a negative marker. The samples were then resuspended for further analysis [28, 29]. (supplementary materials, Sect. 4.5)

## MiRNA-21-5p expression

The expression of miRNA-21-5p was assessed in characterized CSCs and different types of MSCs primarily isolated and cryopreserved at CERRMA stem cell banks. Briefly, total RNA including miRNAs was extracted from different cell types using miRNeasy kit (Qiagen, Hilden, Germany) according to the manufacturer’s recommendations. The concentration and purity of RNA (OD260/OD280) were determined by a UV-Vis nanodrop spectrophotometer. Single-stranded cDNA was synthesized using specific primers from TaqMan MicroRNA Assays (ID: 002493). MiRNA-21-5p was assayed using TaqMan MicroRNA Assays #4,427,975 using Bio-Rad CFX96™ Real-Time PCR detection system. The relative miRNA expression was calculated using the comparative threshold (CT) method (2^–ΔΔCT^) after normalization for the expression of U6 as an endogenous control. All reagents were obtained from Applied Biosystems, USA [24, 30]. (supplementary materials, Sect. 5)

## Isolation and culturing of CSCs

CSCs were isolated, purified, and cultured according to a previously established protocol with some modifications [31]. Briefly, six BALB/c mice were anesthetized and heart-exposed. The aorta was cannulated for retrograde perfusion with a basic buffer, which contains MEM, HEPES, taurine, and glutamine. The heart was then digested with collagenase solution, blocked with BSA solution, and digested tissue and cells were separated by centrifugation. CSCs were purified from CD45^+^ cardiac mast cells using EasySepTM Mouse CD45 positive selection kit (Stem cell Technology cat# 18757) following the manufacturer protocol. The cells were cultured in growth media equal mixture of DMEM-F12-Ham’s (supplemented with 1% of both insulin-transferrin-selenium, P/S-fungizone, and 0.1% gentamicin) and neurobasal medium (supplemented with 1% l-glutamine, 2% B27 supplement, and 1% N2 supplement from Invitrogen, cat# 17502-048). Then, the following factors were added to the media mixture, including 10% FBS, epidermal growth factor (20 ng/mL, Peprotech, cat# 100 − 15), basal fibroblast growth factor (10 ng/mL, Peprotech, cat# 100-18B), and leukemic inhibitory factor (10 ng/mL, Millipore, cat# LIF2010).

## Characterization of CSCs

### Immunofluorescent staining

Once confluent, CSCs were washed, fixed, permeabilized, blocked, and immunofluorescently stained using GATA4 primary rabbit monoclonal antibodies and goat anti-rabbit secondary antibodies. The cells were visualized using a confocal microscope and incubated for 45 min [32]. (supplementary materials, Sect. 7.1)

### CSCs surface characterization

CSCs cell surface markers were assessed using anti-CD45-PE anti-CD90-fluorescein isothiocyanate, anti-CD117-PE conjugated antibody, anti-CD105, anti-CD73, anti-CD Sca1, and anti-OCT4, followed by secondary antibody staining with anti-mouse IgG conjugated antibody. A FACS Calibur flow cytometer and Cell Quest software were used to analyze the fluorescence of viable cells [33]. (supplementary materials, Sect. 7.2)

## Experimental design and grouping

Following validation of the CSCs populations, cultured CSCs were seeded in 6-well plates and divided into four groups: *control group*; CSCs were grown under standard incubator conditions in 5%CO2 and 21% oxygen in full growth media, *oxygen-glucose deprivation (OGD)*; CSCs in this group were exposed to 1% oxygen in a hypoxic chamber (stem cell technologies, 27310) for 24 h and grown in media consisting of DMEM without glucose or pyruvate and immediately harvested for assessment following the 24 h, *OGD/reoxygenation (OGD/R);* CSCs were exposed to the same conditions of hypoxia and glucose deprivation for 24 h, however, were replenished with full growth media and standard culture conditions for 72 h before harvesting, and *sEVs treated group*; CSCs in this group were exposed to hypoxia and glucose deprivation for 24 h and replenished with full growth media supplemented with sEVs (35 µg). Each assay was performed in triplicate. The conditioned media from all groups was collected for oxidative stress testing. Additionally, Sca-1+/CSCs were grown in chamber flasks for immunofluorescence staining.

## Induction of ischemia-reperfusion injury

Oxygen deprivation was induced using a hypoxic chamber (stem cell technologies, 27310) to simulate hypoxia by creating a self-contained, sealed, and low-oxygen environment. Per manufacturer protocol, the hypoxic chamber was purged with a gas mixture of 1% O2 and 5% CO2 balanced with nitrogen at a rate of 20 L/min for 5 min [34]. (supplementary materials)

## Assessment

### Hypoxia-induced factor-1α

After 24 h for the OGD group and 72 h for OGD/R and sEVs groups, cells were washed and fixed with 4% PFA for 10 min at 37 °C, permeabilized using 0.1% Triton X-100 for 15 min at room temperature, then blocked for 1 h using 2% BSA and finally incubated primary monoclonal anti- HIF-1α ab [H1alpha67] (ab1). Suspended cells were then labeled with anti-mouse Alexa-Fluor^®^ 555 dye for analysis using a BD FACS Calibur flow cytometer. While cells grown on chamber slides were used for immunostaining of hypoxic cells using HIF-1α. Control normoxic cells were kept under standard incubator conditions. Following fixation, permeabilization, and blocking, cells were stained with HIF-1α ab [H1alpha67] (ab1) 1:100 at 4 °C overnight. The following day, primaries were washed and cells were labeled with Goat anti-mouse secondary (life technology A21422) in 1:200 dilutions for 45 min at room temperature, and DAPI was added as a counterstain with the secondary antibodies in a dilution of 1:1000. Visualization of the immunofluorescent-stained cells was through the confocal microscope (Leica TCS SP5, Germany). *Hif-1α* gene expression was also assessed using RT-qPCR, as detailed in the supplementary file (Sect. 9.1.B).

### Cell proliferation

#### Cell cycle assay

Cell cycle assay was performed by PI/RNase reagent (cat# 4087 S, Cell Signaling, Life Technology, USA). Briefly, cells were centrifuged, washed, and resuspended in methanol. Methanol-fixed cells were centrifuged and resuspended in PBS to wash excess methanol. Washed cells were centrifuged and resuspended in PI/RNase reagent. The cells were then incubated in the dark for 15 min, and the stained cell suspension was analyzed using BD FACS Calibur flow cytometry. (supplementary materials)

#### Proliferation marker Ki67

Cells were fixed with 4% PFA for 10 min at 37 °C, permeabilized using 0.1% Triton X-100 for 15 min at room temperature, then blocked for 1 h using 2% BSA and finally incubated with the Alexa Flour 488-conjugated anti-rabbit Ki67 antibody (IgG, Cell Signaling Technology, USA, cat# 11882 S). Assessment and data analysis were done using BD FACS Calibur flow cytometry.

#### EdU DNA staining

EdU staining was carried out with Click-iT™ EdU Alexa Fluor™ 594 Flow Cytometry Assay Kit (cat# C10420, Life Technologies, 29851 Willow Creek Road, Eugene, USA) according to the manufacturer protocol. EdU was added to the culture medium and mixed well. Cells were harvested, washed, and incubated with Click-iT fixative at RT in the dark. Cells were washed and incubated with Click-iT^®^ saponin-based permeabilization, and wash reagent. The EdU reaction cocktail was then incubated with cells for 30 min. Cells were washed with 1% BSA and another wash, and EdU staining was analyzed using flow cytometry. (supplementary materials)

### Apoptosis

Cell pellets from all groups were washed in PBS and resuspended in 100 µL Annexin V binding buffer (1x). Then, Annexin V Alexa Fluor 488 (5 µL, BD Biosciences Pharmingen TM, USA) and PI (5 µL) were added for 15 min incubation in the dark at room temperature. Annexin/PI-stained cells were evaluated according to the previously described method on a BD FACS Calibur instrument (Becton Dickinson, USA) fitted with a 488 nm argon laser. A minimum of 10,000 cells per sample were acquired and analyzed using Cell Quest Pro software.

### Oxidative stress index (MDA/TAC index)

Malondialdehyde (MDA), the lipid peroxidation marker [35], and total antioxidant capacity (TAC) [36] were measured in cell culture media using a spectrophotometer (supplementary materials, Sect. 11). A ratio of MDA (nmol/mL) to TAC (uM/L), known as the MDA/TAC oxidative stress index, was then calculated to determine the oxidative stress status [37]. 

## PTEN /Akt/ pAkt/ HIF-1α pathway

The PTEN/Akt/pAkt/HIF-1α pathway, a downstream target of miRNA-21-5p silencing, was investigated by assessing the protein expression by western blot. Total proteins were extracted from CSCs, quantified, and separated using SDS-PAGE. After blotting, nitrocellulose membranes were incubated with primary antibodies (Cell Signaling Technology) against PTEN, AKT, Phospho-Akt, and β-actin. Then, membranes were incubated with an anti-rabbit IgG antibody followed by chromogenic western blot band detection. Band density was assessed using BIO RAD Gel DocTM XR with Image Lab software for band imaging and densitometry. (supplementary material, Sect. 12)

## Statistical analysis

Statistical analyses were performed by GraphPad Prism version (5v). All data are shown as mean ± SD derived from the biological triplicates. Determination of the statistical differences between groups was by Student t-test (two groups), one-way ANOVA followed by multiple Tukey comparisons (3 or more groups), or two-way ANOVA followed by Tukey multiple comparisons (grouped data). For all statistical tests, *p* < 0.05 was considered statistically significant.

## Results

### Characterization of BMMSCs and sEVs with a robust miRNA-21-5p expression

Primary cultured BMMSCs showed a monolayer of spindle-shaped cells that reached 80% confluency after 12 days. (Supp. Figure 1A and B).

BMMSCs successfully differentiated into osteoblasts, chondrocytes, and adipocytes, noted by the characteristic positive staining of each cell lineage with their corresponding special stains; Ca deposits with Alizarin stain; collagen-specific proteoglycans with Alcian blue; and fat droplets with Oil red stain, respectively (Supp. Figure 1C).

Immunophenotyping of BMMSCs at P3 showed high expression of the mesenchymal surface markers; CD90 (97.02%), CD44 (96.07%), CD105 (95.5%), and CD73 (96.06%), whereas the cells showed low expression of hematopoietic stem cell markers CD45 and CD11b, showing 0.74% and 0.1%, respectively (Supp. Figure 1D).

**Fig. 2:**
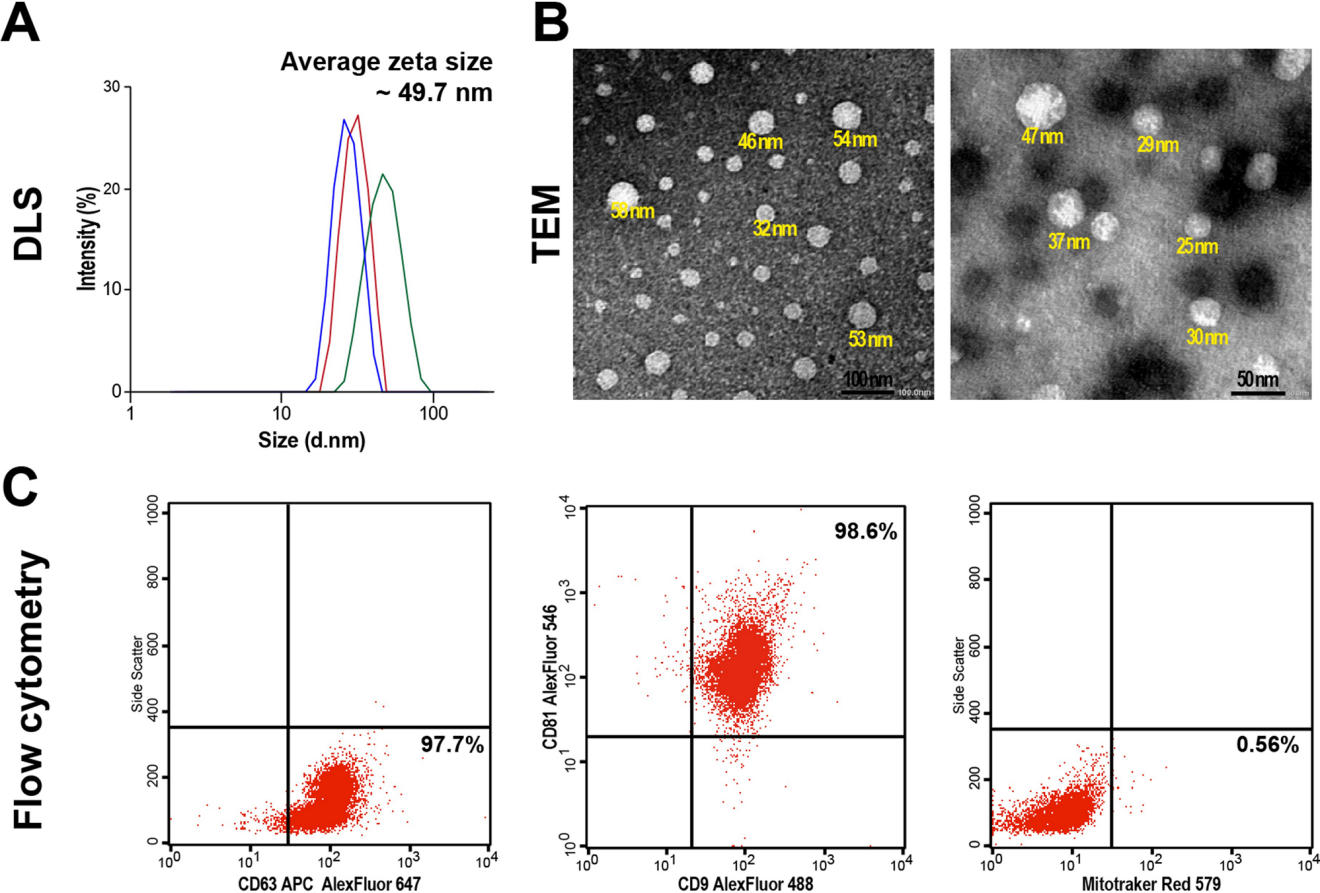
Characterization of BMMSCs-extracted sEVs. **(A)** The size of the isolated sEVs by the dynamic light scattering (DLS). The intensity analysis curve shows the average peak size of sEVs to be ~ 49.7 nm. **(B)** The TEM (120 kV) confirms the presence of sEVs with a heterogeneous size, ranging from 18 to 50 nm (scale bars 200 and 50 nm, respectively). **(C)** The flow cytometric analysis of fluorescently labeled sEVs shows 97.7% of CD63^+^, 98.6% of CD81^+^/CD9^+^, and negatively mitotracker-stained vesicles. The representative flow cytometry plots are among triplicate runs, with the number of acquired vesicles per single flow reading equal to 5000 events

After confirmation of BMMSCs lineage, the isolated sEVs were further characterized. The average zeta size of isolated sEVs was 49.7 nm (Fig. 2A). Additionally, TEM revealed the isolated sEVs were rounded structures with an evident lipid bilayer with various sizes ranging from 25 to 58 nm (Fig. 2B).

The total protein content of sEVs was quantified using a BCA assay with a mean of 675 µg/ml. Moreover, the phenotyping of sEVs revealed 97.7% of CD63 expression and a CD9 and CD81 double-expressing population of 98.6%. The mitochondrial fluorescent dye was almost undetected (0.54%), further robustly validating the sEVs population isolated (Fig. 2C).

The RT-qPCR results showed that the relative miRNA-21-5p expression in the isolated BMMSCs was the highest amongst other MSCs (AmMSCs and ATMSCs; *p* < 0.001 and *p* < 0.0001, respectively), with the lowest level being in CSCs (Supp. Figure 2).

## Characterization of Sca-1+ /CSCs population

**Fig. 3:**
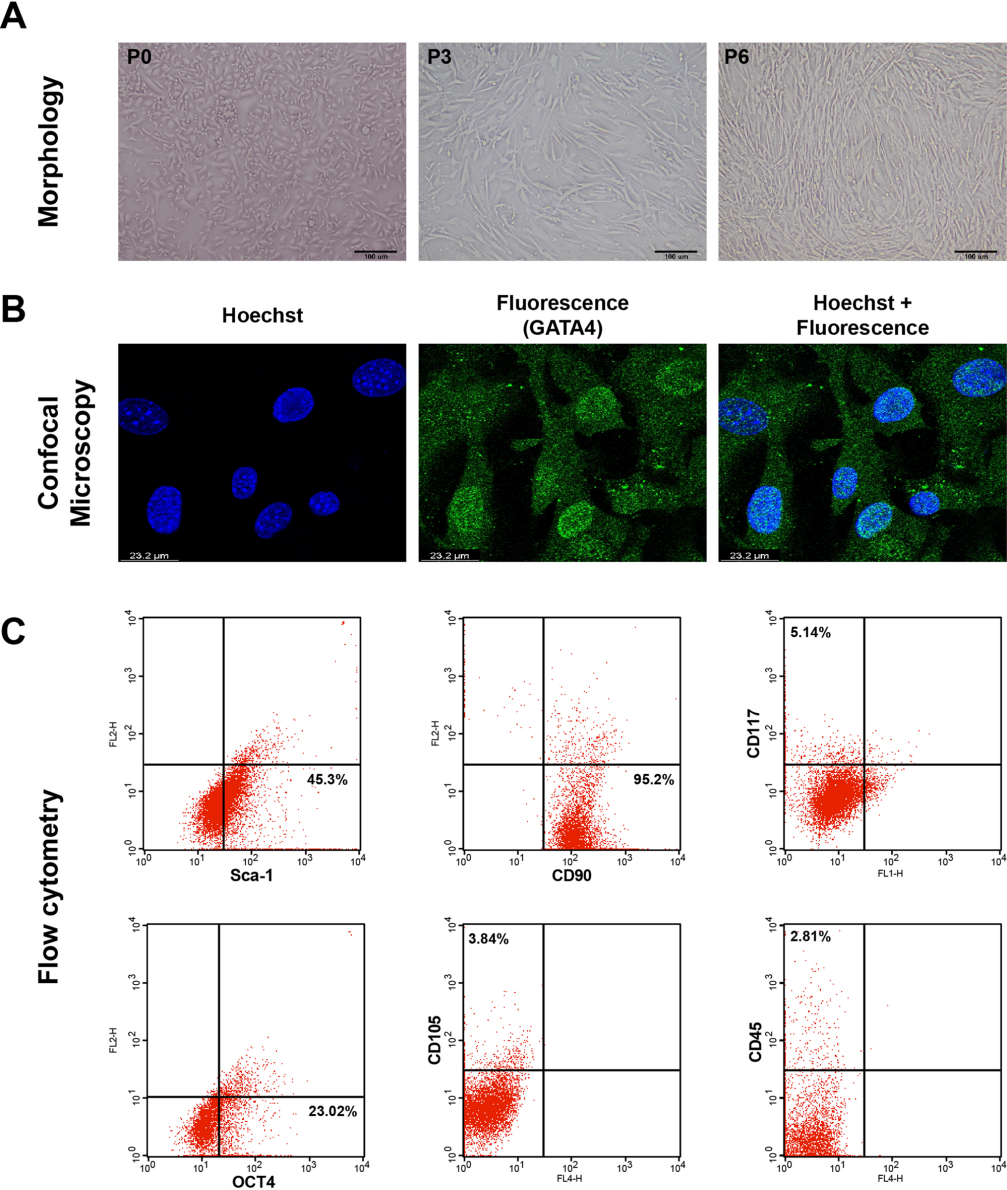
Characterization of CSCs/Sca-1^+^. **(A)** Representative phase contrast microscopic images of CSCs (scale bar 100 μm) at passages P0, P3, and P6, with 90% confluence from an initial seeding density of 25 × 10^4^ CSCs in a six-well plate. **(B)** Confocal microscopy images of CSCs GATA4 cardiac transcriptional factor followed by Hoechst staining (representative of five random confocal microscopic images examined with a scale bar of 23.2 μm). GATA4 fluorescence is green, counterstaining of the nucleus with Hoechst is blue, and overlaid images represent co-localization. **(C)** Flow cytometry assay displays the percentage expression of CSCs markers (Sca1, CD90, CD117, OCT4, CD105, and CD45). The flow cytometer readings were analyzed in triplicate with the acquisition of 10,000 events

Bright-field images of the primary passage (P0) showed heterogeneous CSCs as a mixture of small and round cells, while others showed processes. At P3 and P6, CSCs formed a morphologically homogeneous population of spindle-like cells (Fig. 3A).

Immunofluorescent-labeled CSCs showed positive GATA4 transcription factor colocalized in the cytoplasm with nuclear homing as a site for GATA4 expression. Furthermore, the double staining with Hoechst dye revealed the preservation of the nuclear DNA (Fig. 3B).

Upon testing the expression of stem cell surface and intracellular markers, the flow cytometric data revealed that these cells were positive for Sca-1 (45.3%), CD90 (95.2%), and OCT4 (23.02%). Meanwhile, they were low positive for CD117 (5.14%) and CD105 (3.8%) and almost negative for the hematopoietic stem cell marker CD45 (2.81%) (Fig. 3C).

## Prolonged hypoxia induces HIF-1α overexpression in Sca-1+/ CSCs cells

**Fig. 4:**
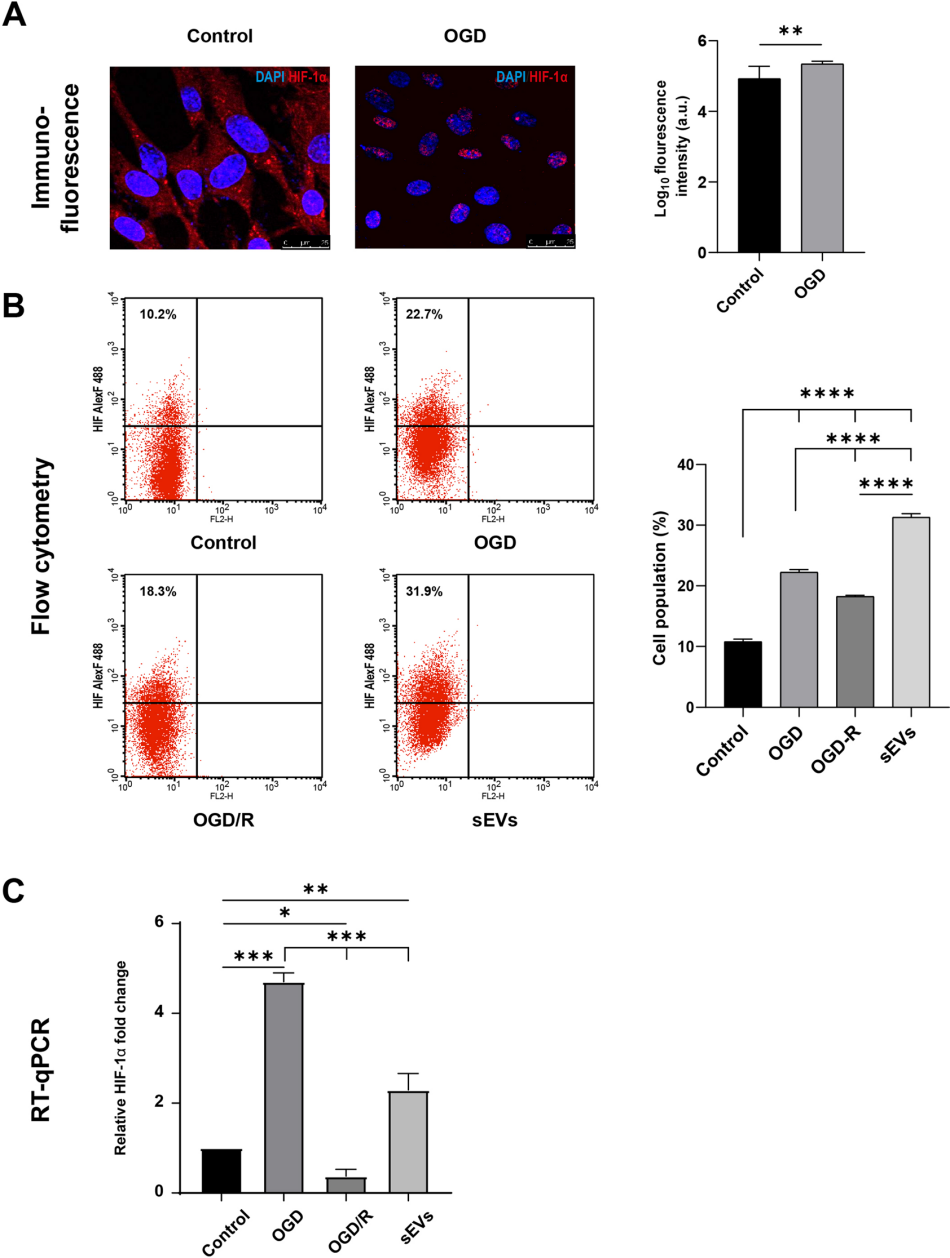
HIF-1α expression in the sEVs CSCs/Sca-1^+^ group in different experimental groups.**(A)** Fluorescent microscopic images (scale bar 25 μm) show the HIF-1α immunofluorescent staining of CSCs/Sca-1^+^ under control normoxic conditions and OGD, with significant** (*p* < 0.01) visible signal in OGD-CSCs as documented in the bar chart of the immunofluorescent analysis. **(B)** The representative dot plots of flow cytometric analysis of HIF-1α expression reveal its percentage in the gated cells of untreated and treated groups, where sEVs- CSCs/Sca-1^+^ express the HIF-1α significantly**** higher than the control as displayed in the associated graph bar. **(C)** The bar chart of the gene expression analysis *of Hif-1α* mRNA RT-qPCR. Relative mRNA expression of *Hif-1α* in control, OGD, OGD/R, and sEVs CSCs/Sca-1^+^ groups. The results are presented as fold change relative to the control group, and data are normalized against *Gapdh.* The data in all bar charts are the mean ± SD of three independent experiments, each performed in triplicate (from 25 × 10^4^ CSCs/Sca-1^+^ seeded into three wells of six-well plates). Unpaired t-test was used to analyze fluorescence intensity and one-way ANOVA with Tukey’s multiple comparisons for flow cytometry and RT-qPCR reveals *of *p* < 0.05, **of *p* < 0.01, ***of *p* < 0.001, ****of *p* < 0.0001while ns denotes non-significant of *p* > 0.05

For the OGD model verification, the immunofluorescence staining of Sca-1+/ CSCs exposed to OGD with anti-HIF-1α antibody revealed a significant intense nuclear fluorescent signal compared with control Sca-1+/ CSCs exposed to normoxic conditions (*p* < 0.01, Fig. 4A). Using flow cytometry, sEVs-treated cells showed a statistically significant increase in HIF-1α expression (31.9 ± 0.57%) in comparison with the control (10.2 ± 0.45%, *p* < 0.0001). The HIF-1α increase in OGD (22.7 ± 0.4%) and OGD/R (18.3 ± 0.2%) cells was significant to the control cells (*p* < 0.0001). The highest HIF-1α expression was observed in the reoxygenated Sca-1^+^/ CSCs with sEVs compared with the other treated groups (*p* < 0.0001, Fig. 4B). Induction of hypoxia was further validated by the relative mRNA expression of HIF-1α in the OGD- Sca-1^+^/ CSCs group, which was significantly higher (~ 4-fold increase) compared with the control, OGD/R, and sEVs- Sca-1^+^/ CSCs groups (*p* < 0.05, Fig. 4C).

## Reoxygenation/sEVs treatment increased the proliferative capacity of Sca-1+/ CSCs

**Fig. 5:**
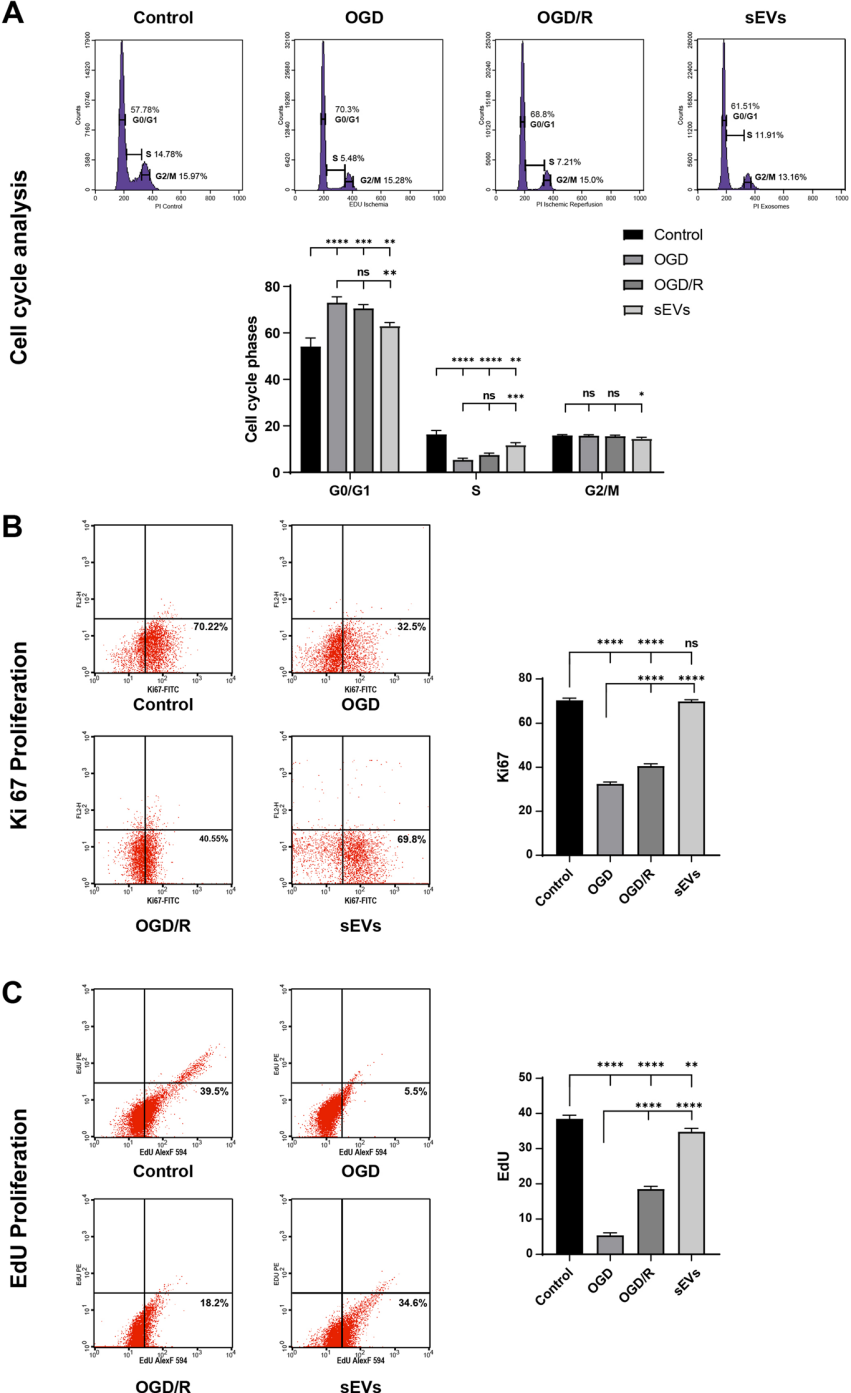
Reoxygenation/sEVs treatment increased the proliferative capacity of CSCs/Sca-1^+^. **(A)** Cell cycle analysis of different CSCs/Sca-1^+^ groups detected by PI/RNase staining with the representative DNA histograms and bar chart displays the distribution and percentages of cell cycle phases. **(B and C)** Representative dot plots of the flow cytometric expression of proliferative Ki67 and EdU antigens staining incorporation in CSCs/Sca-1^+^ showing the expression percentage of the gated cells exposed to OGD and reoxygenation conditions with the corresponding bar charts of the percentage of cell populations of the different reoxygenated groups relative to control and OGD groups. Each graph represents the mean ± SD from three independent experiments; each experiment consisted of triplicates per treatment group, group (each replica consists of 25 × 10^4^ CSCs/Sca-1^+^ seeded into three wells of six-well plates). The significance is marked by *p < 0.05, **p < 0.01, ***p < 0.001, and ****p < 0.0001, while *p* > 0.05 denotes non-significance (ns) from two-way ANOVA for cell cycle analysis and one-way ANOVA for Ki67 and EdU analyses followed by Tukey’s multiple comparisons test in all analyses

The cell cycle phase distribution of Sca-1^+^/ CSCs exposed to OGD and reoxygenation models depicted in Fig. 5A reveals that the cell cycle arrest is more evident in the OGD model, where there is a significant increase in cell population in the G0/G1 phase (73.0 ± 2.5%, *p* < 0.0001). A concomitant slowdown of the DNA synthesis was evident upon oxygen deprivation of the OGD-CSCs, with a significant decline in the S phase cell population compared with control (5.3 ± 0.67%, *p* < 0.0001) and a similarity in the G2/M phase cell population (15.7 ± 0.41, *p* > 0.05). On the other hand, Sca-1^+^/ CSCs treated with sEVs were predominantly in the S phase (11.8 ± 0.9%) relative to the OGD group (5.3 ± 0.6%, *p* < 0.01) and the OGD/R model (7.5 ± 0.7%, *p* > 0.05) that remained low. Consequently, reoxygenation with sEVs supplementation decreased the cell cycle G0/G1 arrest significantly compared with other reoxygenated groups OGD/R (63.1 ± 1.4% and 70.5 ± 1.6%, respectively, *p* < 0.001).

This proliferative capacity in the sEVs group was assured with Ki67 positive flow cytometric characterization, taking the negative control of untreated CSCs as a gated Ki67- population. After 72 h of sEVs incubation with CSCs, the Ki67 level elevated to 69.7 ± 0.7% which was indistinguishable from the 70.3 ± 0.9% of Ki67 in control Sca-1^+^/ CSCs (*p* > 0.05). Cell proliferation rates of the sEVs group (*p* < 0.0001) were significantly higher than the OGD/R and OGD groups, which showed an expression of 40.5 ± 1.07% and 32.4 ± 0.8% respectively (Fig. 5B).

EdU staining assay displayed increased proliferation in the reoxygenated sEVs group (34.8 ± 0.9%), indifferent to the control group (38.4 ± 1.0%, *p* > 0.05), as shown in Fig. 5C. Conversely, halting the proliferative status of the OGD group at 5.3 ± 0.7% and the OGD/R group at 18.5 ± 0.7% was prominently decreased compared with the control group (*p* < 0.0001). Moreover, the OGD/sEVs group showed a highly significant proliferation rate compared with the OGD and OGD/R groups (*p* < 0.0001).

## Small extracellular vesicles (sEVs) attenuate accelerated cell death in Sca-1+/ CSCs cells

**Fig. 6:**
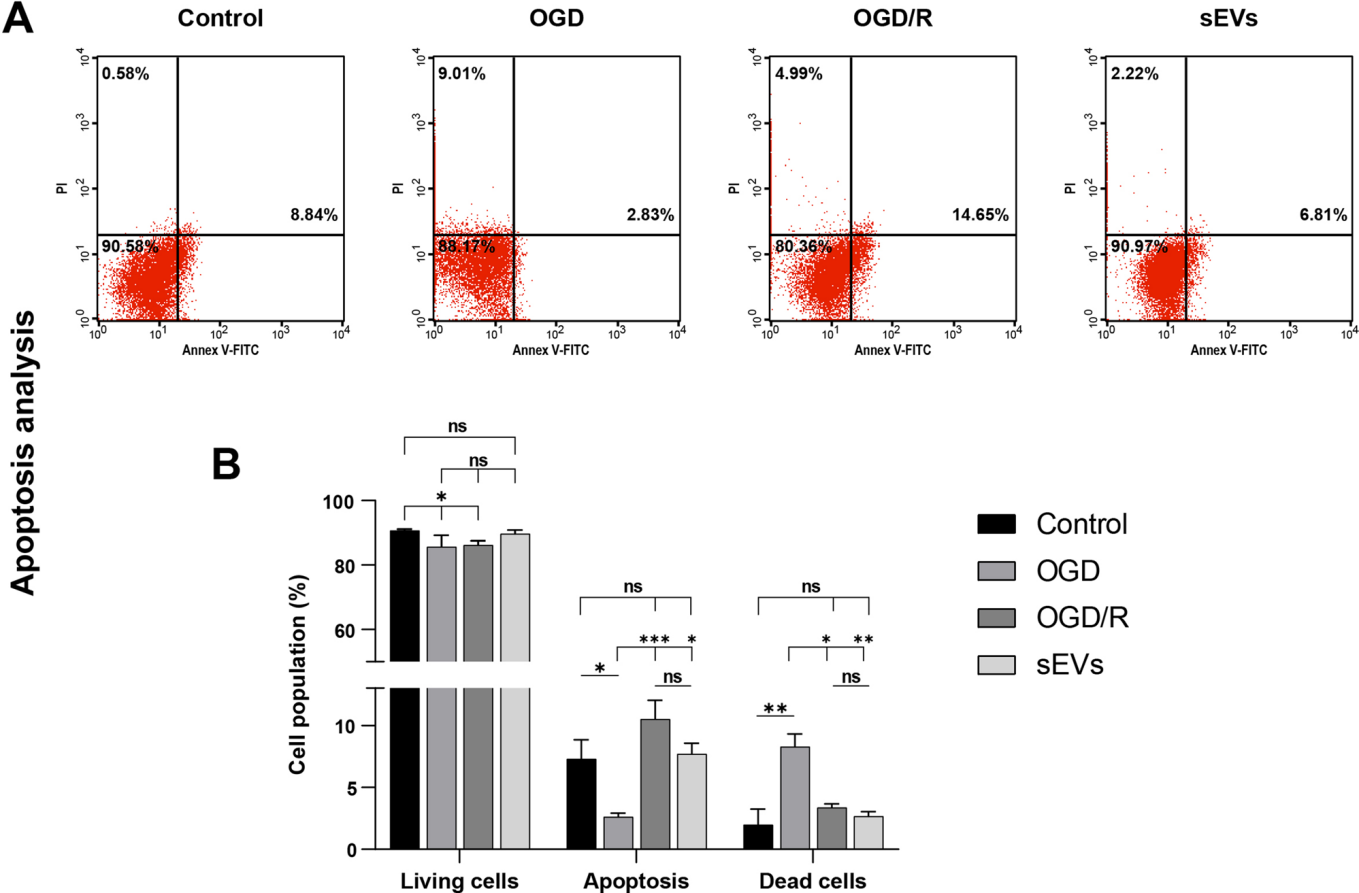
Flow cytometry analysis of apoptosis in CSCs/Sca-1^+^ under three conditions (OGD, OGD/R, and sEVs) in relation to normal cells. **(A)** Representative dot plots of Annexin V/PI expression and the corresponding bar charts **(B)** of different CSCs/Sca-1^+^ groups reveal that reoxygenation*** and sEVs treatment induce significant apoptosis rather than cell death compared with hypoxic CSCs/Sca-1^+^. The data of bar plots are the mean ± SD of three independent experiments, each performed in triplicates for each group (each replica consists of 25 × 10^4^ CSCs/Sca-1^+^ seeded into three wells of six-well plates ), with *p < 0.05, **p < 0.01, and ***p < 0.001, while *p* > 0.05 of non-significance (ns) from two-way ANOVA followed by Tukey’s multiple comparisons test

The oxygen deprivation-induced stress lasted for 24 h and induced significant necrotic cell death, 8.2 ± 1.03%, *p* < 0.01 compared to the control, as compared with the apoptotic pathway (2.6 ± 0.3%). Upon oxygenation, the rate of apoptotic cell death significantly increased in the OGD/R (10.5 ± 1.5%) and sEVs (7.7 ± 0.8%) compared with the OGD (*p* < 0.001 and *p* < 0.05, respectively). However, the rate of apoptosis in sEVs treated cells was not significant from controls (*p* < 0.05). (Fig. 6).

## Small extracellular vesicles (sEVs) alleviate post-hypoxia reoxygenation-induced oxidative stress

**Fig. 7:**
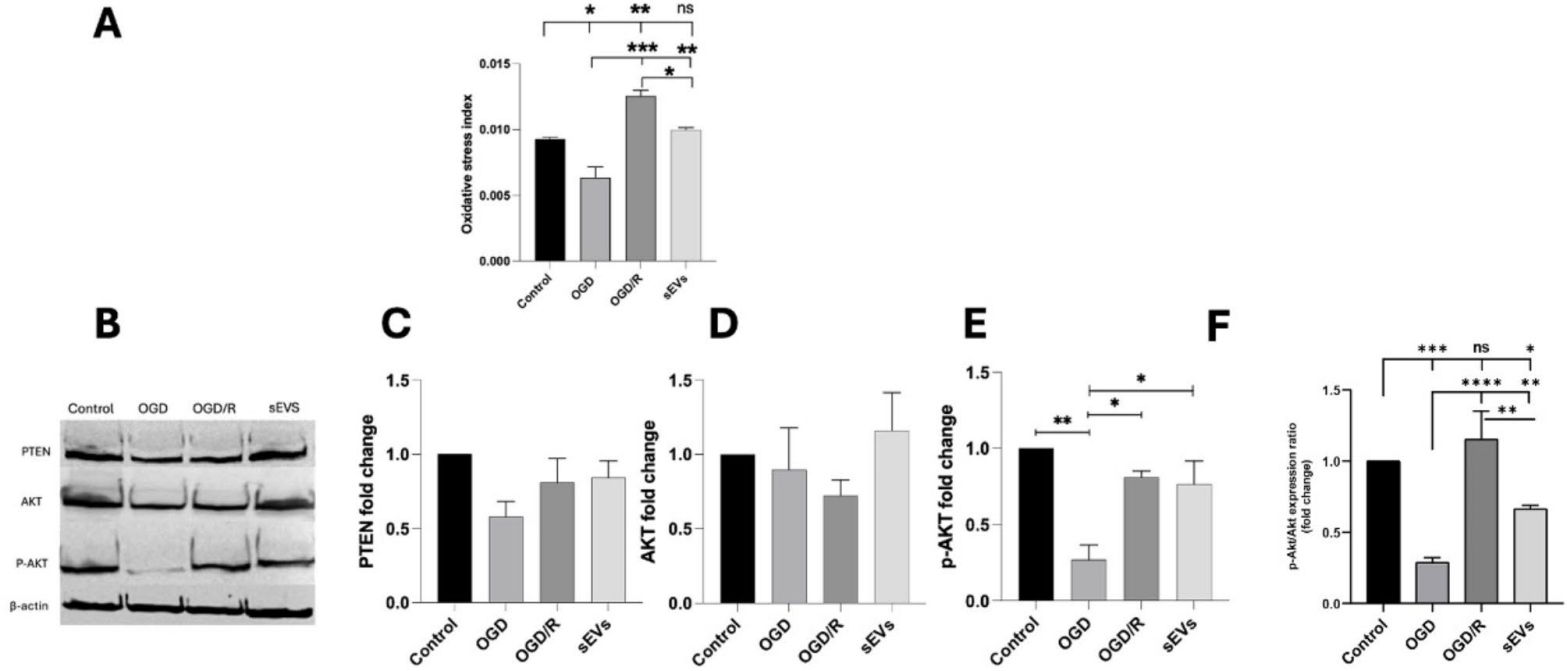
Oxidative stress index and PTEN /AKT/p-AKT expression in the CSCs/Sca-1^+^ groups. Oxidative stress is indicated by the overall oxidative stress index (**A**). The representative western blots and the corresponding densitometric quantification show PTEN 54kDa (**B**, **C**), AKT 60 kDa (**B**, **D**), p-AKT 60 kDa (**B**, **E**), the ratio of p-AKT to AKT (**F**). and β -Actin 45 kDa (**B**) in control, OGD, OGD/R, and sEVs CSCs groups. β-actin is the loading control. Data in each bar chart are representative of mean ± SD of three independent experiments performed in triplicates each (each replica consists of 25 × 10^4^ CSCs/Sca-1^+^ seeded into three wells of six-well plates), where one-way ANOVA followed by Tukey’s multiple comparisons test reveals *p < 0.05, **p < 0.01, and ***p < 0.001, while ns means non-significance of *p* > 0.05

Relieving oxidative stress was proportionally related to the reoxygenation way, where sEVs supplementation during the reperfusion model revealed potent antioxidative potentiality (Fig. 7A, supplementary Fig. 1). In the SEVs group, sEVs shifted the redox balance towards the normal, recording 0.009 ± 0.00 for both sEVs and control groups (*p* > 0.05) (Fig. 7A). Meanwhile, reoxygenation in the OGD/R group loaded the CSCs with a significant oxidative burden up to 0.012 ± 0.00 (*p* < 0.001) compared with the OGD group, (Fig. 7A).

## Effects of small extracellular vesicles (sEVs) on the PTEN/pAkt/HIF-1α pathway

PTEN protein expression was not significantly different between all groups (*p* > 0.05 in all groups). The OGD and OGD/R groups showed a decrease in Akt expression, though it was not significant (*p* < 0.05). There was a slight increase in Akt expression in the sEVs CSC group, although non-significant (*p* = 0.4). On the other hand, the level of pAkt expression in the OGD was significantly lower than the control, OGD/R, and sEVs groups (*p* < 0.05). Similarly, the ratio of p-AKT to AKT relative protein expression was significantly lower in the OGD than in the control, OGD/R, and sEVs groups (* ** *p* < 0.01, * ** *p* < 0.01, and* * *p* < 0.05 respectively)(Fig. 7B-F).

## Discussion

Ischemic injury leads to cardiomyocyte death, which eventually results in heart failure. While post-ischemic reperfusion revascularizes the injured myocardium, it also increases oxidative stress, leading to further cardiomyocyte death and necrosis. This dilemma is an economic and health burden worldwide [38]. Several processes govern this hypoxic and reperfusion injury in the heart cardiomyocytes, including apoptosis, cell cycle modulation, fibroblast remodeling, and HIF-1α stabilization [39]. 

In the current study, we isolated a pool of cardiac progenitors from adult mouse hearts that showed Sca-1^+^, CD90^+^, GATA4^+^, OCT4^+^, CD105^+^, low CD177^+^, and CD45^−^ expressions. Upon in vitro expansion, the isolated cells showed typical mesenchymal morphology and growth features consistent with the previously reported mouse Sca-1^+^ cell population [4, 31, 40]. Sca-1-mediated signaling is crucial for CSC development, and its beneficial effect might be involved in the responses to hypoxic and ischemic conditions [41]. A recent study suggested that adult epicardial-derived CSCs are enriched in Sca-1, suggesting that this subpopulation increases postnatally and is of importance in adults [42]. In vitro studies showed that Sca1^+^ cells express cardiac genes and proteins upon cardiogenic stimulation [43]. Mouse Sca-1^+^CD31^−^ cells were previously proven to be multipotent and could differentiate into cardiac cell lineages when primed with certain miRNAs [44]. We further validated the differentiation potential of this population into cardiac lineages in consequent studies (data not shown).

In our study, we sought to focus on the in vitro behavior of Sca1+/CSC exposed to hypoxia followed by reoxygenation with or without sEVs in terms of the proliferation rates, apoptosis, oxidative stress, and phases of the cell cycle. Herein, we showed that hypoxia increased cell cycle arrest in the Sca-1^+^/CSCs, as shown by the increased population in the G0/G1 phase. The cell cycle arrest continued despite reoxygenation in the OGD/R group yet decreased in the sEVs-treated group with a concomitant increase in the S phase, suggesting that hypoxia for 24 h in Sca-1^+^/CSCs created a state of maintained quiescence that was persistent after reoxygenation. In the presence of sEVs, Sca-1^+^/CSCs started shifting to the S phase and DNA synthesis. We propose that sustained cell cycle arrest during reoxygenation is a protective mechanism against CSCs dividing in a ROS-rich environment. Exclusively, in the presence of sEVs and their enhanced antioxidant effects, CSCs restored their proliferative capability. Our study suggests that hypoxia followed by reoxygenation are insufficient to activate quiescent Sca1+/CSCs, while sEVs provide essential reinforcement to protect the Sca1+/CSC from oxidative stress and enable cell division. Although hypoxia induces cell death in various cell types, it is a well-known favorable condition for the growth of stem cells and was shown to enhance in vitro proliferation, differentiation, and survival of embryonic and neural stem cells [45]. The exact molecular and secretome conditions that regulate such responses in CSCs have not yet been elucidated. We sought to test the response of Sca1+/CSCs to hypoxia and whether they share this property with stem cells.

We further examined if the sEVs from BMMSCs overexpressing miRNA-21-5p were related to the observed behavior of Sca-1^+/^CSCs cells under OGD and OGD/R. sEVs isolated from BMMSCs showed typical rounded morphology, size, and expression of CD 63,9, and 81 tetraspanins. BMMSCs were chosen as the main source of EVs as they showed a higher expression of miRNA-21-5P, the target miRNA in this study when compared with other stem cell sources. We further investigated a downstream target pathway for miRNA-21-5p, the PTEN/pAkt/HIF-1α pathway.

PTEN regulates apoptosis, growth, differentiation, and cell proliferation, with an inverse correlation with cell survival [46]. Neonatal cardiomyocytes overexpressing PTEN showed higher apoptosis, while mutant expression increases cell growth. Phosphatidylinositol (PI)-3,4,5-triphosphate (PIP3) is the activator of Akt [47]. The conversion of PI-4,5-diphosphate to PIP3 is triggered by PI3-kinase (PI3K). The PI3K/Akt signaling pathway regulates a wide range of cellular activity, including cell survival, proliferation, and metabolism [33]. Research has demonstrated that PTEN activity primarily determines PIP3 levels, which regulates Akt activation [48]. Previous studies have established that hypoxia induces a persistent increase in phosphorylation of Akt in cardiac progenitors for up to 24 h, with the effect peaking at six h. The exposure time to hypoxia is crucial to determine the fate of cardiac cells. Short-term hypoxia (4–24 h) leads to overexpression of pAkt, PI3K, and HIF-1α. However, these findings are reversed after 24 h to 7 days of hypoxia, resulting in cell death [49]. 

In our study, although OGD for 24 h did not significantly affect the expression levels of PTEN or Akt, it significantly decreased the expression of pAkt compared to the control group, which was reversed by the IR and sEVs treatment. The most significant post-translational factor influencing Akt activity is phosphorylation. Phosphorylated Akt is essential for cell growth and proliferation, cellular metabolism, and cell survival and apoptosis [50]. 

Additionally, there was a declined proliferation rate and increased cell death, which indicates an overall decrease in the survival of Sca-1 + cells. Our findings suggest that at 24 h, Sca-1+/CSC cells start to switch from pro-survival to cell death signaling and require augmentation to ensure their survival and regenerative potential in vitro. BMMSCs-sEVs enhance Sca-1+/CSC survival through increased expression of Akt and pAkt. Shi and his coworkers have demonstrated similar results using MSC-derived exosomes on CSC exposed to H_2_O_2_ hypoxia besides the decreased apoptosis via the PTEN/PI3K/Akt pathway [33]. 

In the present study, sEVs induced even higher expression of HIF-1α in CSCs, indicating a possible synergistic effect between hypoxia and BMMSCs-derived sEVs-rich in miRNA-21-5p in stabilizing HIF-1α. The HIF-1α pathway chiefly mediates the adaptation to ischemic-induced hypoxia [51, 52]. The effects of HIF-1α stabilization on apoptosis are intricate as seen by numerous contradictory publications claiming that HIF-1α can either trigger or inhibit apoptosis. However, our results showed that the Sca-1+ /CSC expressed low levels of HIF-1α under normoxic conditions but significantly surged when subjected to OGD.

Upon reoxygenation and supplementation with glucose-rich media, Sca-1+/CSC showed an elevated state of oxidative stress with liberation of ROS species that were not adequately counteracted by antioxidants. We show that the heightened oxidative stress was accompanied by accelerated apoptosis. However, supplementing the media with sEVs upon reoxygenation created a balanced redox state. High ROS levels can inhibit cell differentiation in Sca-1+/CSC, suggesting that antioxidants can enhance cardiomyogenesis [53]. However, optimal timing and level of ROS liberation post-ischemic injury require regulation. Novel antioxidant strategies, including modified biomaterials targeting ROS pathways, are needed for efficient engraftment and activity of resident CSCs. Few studies have investigated MSC-derived sEVs’ antioxidant potential under hypoxic conditions [54, 55]. Several preconditioning approaches have previously enhanced the survival of resident CSCs within the hostile ischemic environment [56]. An earlier study linked MSCs’ therapeutic significance to their capacity for engrafting and differentiation and proved the effectiveness of MSC therapy in treating clinical heart failure [57]. However, these effects are usually short-lived, suggesting that paracrine mechanisms are key to the function of MSCs [58]. Upon reoxygenation and re-nourishing of Sca-1^+/^CSC with culture media enriched in BMMSCs-derived sEVs, the survival and proliferation rates increased with declined apoptosis compared with the OGD and OGD/R groups. In line with previous studies [59, 60], we show that BMMSCs-derived sEVs enhanced the proliferation of cultured Sca-1^+/^CSC, their exit from quiescence, and promoted DNA synthesis and mitotic activity. These findings are crucial in the translational field, where sEVs can be delivered early following an ischemic attack to enhance the internal regenerative power of Sca1+/CSCs. A limitation of our study was not being able to test this regenerative power further *in vivo.* We further recommend investigating the transplantation of Sca1+/CSC primed with sEVs or sEVs alone into an animal model of IR to further validate these findings.

Although miRNA-21 has been shown to enhance cardiac fibrosis post-MI in vivo by activation of fibroblasts through several pathways [61], its central anti-apoptotic role has also been shown in several cell types. In previous studies, hypoxia increased the levels of both exosomal- and non-EV miRNA-21-5p via HIFα and posed anti-inflammatory and anti-apoptotic effects on the recipient cells [59, 62, 63]. MiRNA-21-5p levels increase in mouse and in vitro stroke models. We focused on the anti-apoptotic role of sEVs miRNA-21-5p *invitro* in Sca-1+/CSC exposed to OCG/R to observe its exclusive effect on priming Sca-1+/CSC without the exposure to the in vivo miRNA-21-induced fibrosis. Understanding these dual roles is crucial for developing targeted therapeutic strategies that can mitigate the adverse effects [50] of miRNA-21-induced fibrosis while preserving its protective functions.

This study found that post-hypoxia reoxygenation and glucose replacement led to increased ROS production and apoptosis in the OGD/R group. Cell proliferation rates were lower in OGD/R but higher than in OGD, suggesting that reoxygenation failed to rescue CSCs due to accelerated apoptosis. We show, along with similar studies [64, 65] that intervention using BMMSCs-derived sEVs improves the antioxidant state, preventing post-MI complications and enhancing healing pathways. Additionally, we show that low doses of sEVs (35 µg) were sufficient to induce proliferation, decrease apoptosis, and modulate the PTEN/pAkt/HIF-1α in Sca1+/CSC under OGDR conditions. A similar study showed that under OGD circumstances, at least 50 µg is required to promote proliferation and differentiation in neural stem cells, not CSCs [66]. 

One limitation of our study is the focus on miRNA-21-5p without investigating the potential roles of other miRNAs present in BMMSC-derived sEVs and their contributions to the modulation of the PTEN/pAkt pathway. A detailed analysis of the complete miRNA cargo within these sEVs was not conducted, leaving a gap in understanding the relative abundance and potential impact of other miRNAs. Future research should address this by utilizing advanced methods such as high-throughput sequencing or microarray assays to profile and quantify miRNAs comprehensively. Additionally, the absence of loss- and gain-of-function studies to clarify the specific mechanisms through which miRNAs collectively regulate the PTEN/pAkt pathway and downstream targets like HIF-1α is another limitation. Exploring these areas in future studies will enhance our understanding of the broader functional roles of BMMSC-derived sEVs in regulating cellular responses under ischemic conditions.

## Conclusions

Sca-1+/CSC reside in the postnatal human heart for repair upon injury, yet permanent cardiac damage occurs with consequent heart failure when subjected to ischemia. Although the internal repair potential of Sca-1+/CSC is still unclear, activation of these cells is necessary to increase their effectiveness after ischemic events. In this study, sEVs derived from BMMSCs proved to play this role by shifting the cells from their quiescent state to the dividing phase by increasing their proliferative and antioxidant capacity, decreasing their death, and enhancing their reparative power. The investigated sEVs miRNA-21-5p/ PTEN/pAkt/HIF-1α pathway in this study might be a potential pathway that improves the response of CSCs to OGD/R injury.

## Electronic supplementary material

Below is the link to the electronic supplementary material.

Supplementary Material 1

## Data Availability

All raw data and materials are available on demand.

## Abbreviations

AmSCs: amniotic fluid stem cells
ATSCs: adipose tissue stem cells
BCA: bicinchoninic acid assay
BMMSCs: bone marrow mesenchymal stem cells
CSCs: cardiac stem cells
EVs: extracellular vesicles
sEVs: small extracellular vesicles
HIF-1α: hypoxia-inducible factor-1α
HRE: hypoxic responsive element
IHD: ischemic heart disease
MDA: malondialdehyde
MIRI: myocardial ischemia-reperfusion injury
MSCS: mesenchymal stem cells
ODD: oxygen-dependent degradation
OGD: oxygen-glucose deprivation
OGD/R: oxygen-glucose deprivation /reoxygenation
PHD-2: proline-hydroxylase-2
PTEN: Phosphatase and tensin homolog
TAC: total antioxidant capacity
