# Correction: Small extracellular vesicles enhance the survival of Sca-1+ cardiac stem cells against ROS-induced ischemic-reoxygenation injury in vitro

**DOI:** 10.1186/s40659-025-00611-8

**Published:** 2025-05-29

**Authors:** Radwa A. Mehanna, Hagar Elkafrawy, Marwa M. Essawy, Samar S. Ibrahim, Ashraf K. Awaad, Nehal A. Khalil, Marwa A. Kholief, Abeer Sallam, Heba A. Hamed, Mona A. Barkat, Mohamed F. ElKady, Eman H. Thabet

**Affiliations:** 1 Medical Physiology Department, Faculty of Medicine, Alexandria University, Alexandria, 21500 Egypt; 2 Center of Excellence for Research in Regenerative Medicine and Applications (CERRMA), Faculty of Medicine, Alexandria University, Alexandria, 21500 Egypt; 3 Medical Biochemistry Department, Faculty of Medicine, Alexandria University, Alexandria, 21500 Egypt; 4 Oral Pathology Department, Faculty of Dentistry, Alexandria University, Alexandria, 21500 Egypt; 5 Biotechnology Department, Center of Excellence for Research in Regenerative Medicine and Applications (CERRMA), Faculty of Medicine, Alexandria University, Alexandria, 21500 Egypt; 6 Molecular Biology Department, Center of Excellence for Research in Regenerative Medicine and Applications (CERRMA), Faculty of Medicine, Alexandria University, Alexandria, 21500 Egypt; 7 Forensic Medicine and Clinical Toxicology Department, Faculty of Medicine, Alexandria University, Alexandria, 21500 Egypt; 8 Histology and Cell Biology Department, Faculty of Medicine, Alexandria University, Alexandria, 21500 Egypt; 9 Human Anatomy and Embryology Department, Faculty of Medicine, Alexandria University, Alexandria, 21500 Egypt; 10 Medical Biophysics Department, Faculty of Medicine, Alexandria University, Alexandria, 21500 Egypt

**Correction: Biological Research (2025) 58:12** 10.1186/s40659-025-00593-7

In this article Fig. 7 appeared incorrectly and has now been corrected in the original publication. For completeness and transparency, the old incorrect versions are displayed below.

Incorrect Fig. 7
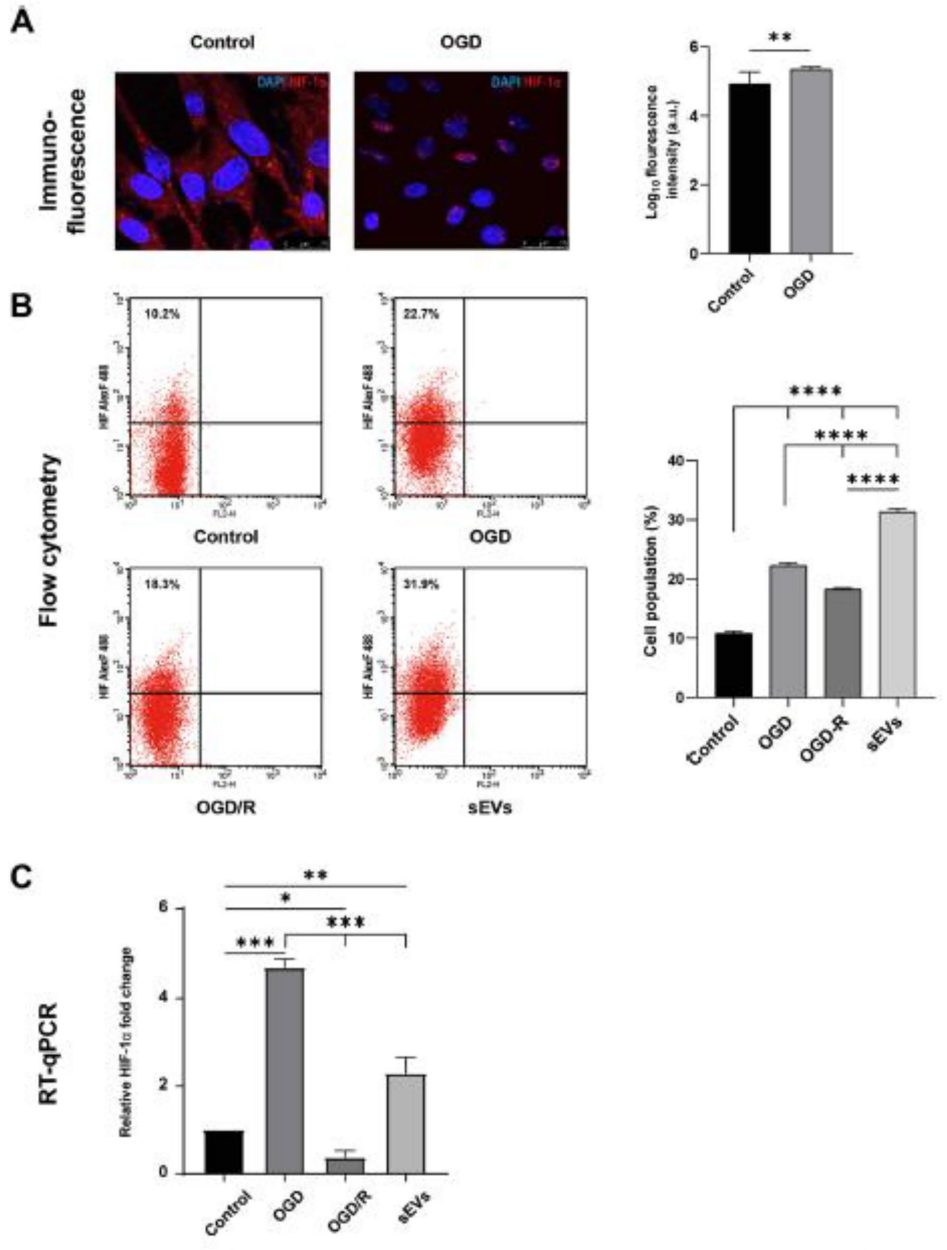


Correct Fig. 7

**Fig. 7:**
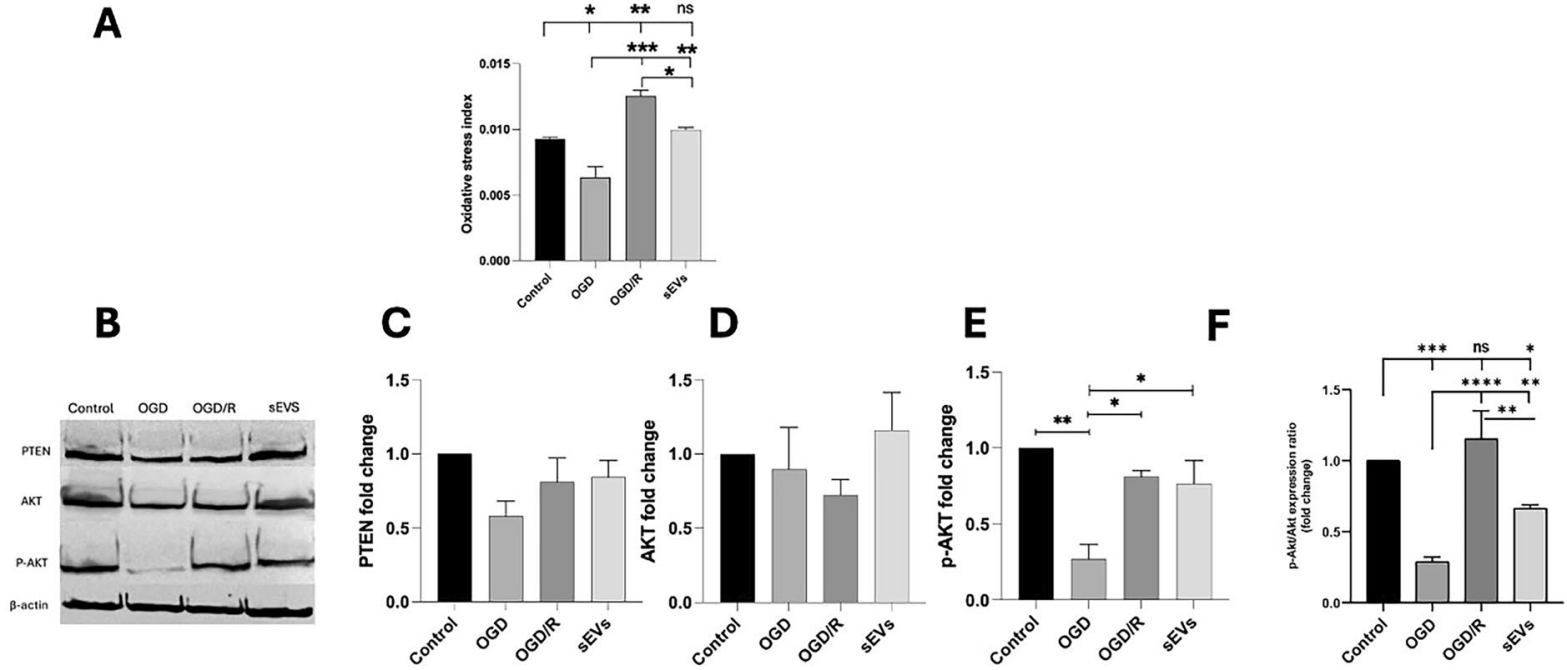
Oxidative stress index and PTEN /AKT/p-AKT expression in the CSCs/Sca-1^+^ groups. Oxidative stress is indicated by the overall oxidative stress index (**A**). The representative western blots and the corresponding densitometric quantification show PTEN 54kDa (**B**, **C**), AKT 60 kDa (**B**, **D**), p-AKT 60 kDa (**B**, **E**), the ratio of p-AKT to AKT (**F**). and β-Actin 45 kDa (**B**) in control, OGD, OGD/R, and sEVs CSCs groups. β-actin is the loading control. Data in each bar chart are representative of mean ± SD of three independent experiments performed in triplicates each (each replica consists of 25 × 10^4^ CSCs/Sca-1^+^ seeded into three wells of six-well plates), where one-way ANOVA followed by Tukey’s multiple comparisons test reveals *p < 0.05, **p < 0.01, and ***p < 0.001, while ns means non-significance of *p* > 0.05

The original article has been corrected.

