# Supplementary material for: Small extracellular vesicles enhance the survival of Sca-1+ cardiac stem cells against ROS-induced ischemic-reoxygenation injury in vitro
Source: Biol Res. 2025 Mar 5;58:12. doi: 10.1186/s40659-025-00593-7 (PMC11881436; doi:10.1186/s40659-025-00593-7)
Supplement: Supplementary file 1 — Supplementary Material 1 [file 40659_2025_593_MOESM1_ESM.docx]

**Supplementary Material**

**Methods**

**1.Isolation and characterization of BMMSCs**

Under sterile conditions, BMMSCs were isolated from femur and tibia of Sprague Dawley Albino rats (3 weeks old and 35-40 g), where their bone marrow was flushed with 5 mL complete culture medium (CCM) and passed through a 70 µm cell strainer and the flow through was centrifuged at 1200 rpm for 5 min. The pellet was resuspended in CCM (low glucose Dulbecco’s modified Eagle’s medium (LG-DMEM, 1.0 g/L glucose; Lonza) supplemented with 10% fetal bovine serum (FBS, Biowest), 2mM L-glutamine, and 1% penicillin/streptomycin (P/S, 10,000 IU/mL/ 10,000 µg/mL, Lonza) then cultured in T25 flask and incubated in 5% CO_2_ incubator at 37°C. After 48 h, adherent cells were washed with phosphate-buffered saline (PBS) with the removal of the non-adherent cells. The CCM was changed every 2–3 days. Upon 90% confluency, the cells were detached with 0.25% trypsin/ethylene diamine tetra-acetic acid (EDTA, Thermo Fisher Scientific, Waltham, MA) solution and split in a ratio 1:3. BMMSCs were monitored by a phase-contrast inverted microscope equipped with a digital camera (Olympus CKX41SF, Japan), until reaching passage three (P3). (1)

**2. Characterization of BMMSCs**

**2.1** **Colony-forming unit assay**

At passage 3, the colony-forming potential (CFU) of the BMMSCs was tested. In this assay, 100 cells were plated on a 60 mm culture dish in CCM and incubated for 14 days. The cells were washed, fixed, and then stained using crystal violet (Sigma-Aldrich, USA) at 3% (w/v) in methanol for 5 min at room temperature. After stain removal, the cells were washed with distilled water. The CFU was calculated as follows: Plating efficiency = (the number of colonies formed/number of cells plated) × 100. All visible colonies were counted, and the colonies displaying five or more cells were scored under the phase-contrast inverted microscope. A CFU of over 40% was optimal for BMMSCs culture. (2)

**2.2** **Differentiation assays**

*In vitro osteogenic differentiation:* cells were grown until 80% confluence and then incubated in an osteogenic induction medium for 21 days (DMEM, 10% FBS, 1% P/S, 1% glutamine, 0.1 µM dexamethasone, 10 mM β-glycerophosphate disodium salt hydrate, and 50 µM L-ascorbic acid 2-phosphate magnesium salt hydrate; all from Sigma-Aldrich, USA). The medium was changed every two days. After induction for 21 days, cells were fixed in 4% PFA for 15 min and stained with Alizarin Red Stain (Sigma-Aldrich, USA) for 30 min at 37°C for imaging the bright orange-red cells using an inverted microscope. (3)

*In vitro chondrogenic differentiation:* with a density of 2.5 × 10^5^ at P3, BMMSCs were resuspended in chondrogenic induction media (StemXVivoVR Chondrogenic Base Media and StemXVivo Chondrogenic Supplement; both from R&D Systems, USA) and then centrifuged at 200 × g for 5 min in a conical falcon tube. The tube cap was loosened to allow gas exchange and was incubated upright at 37°C and 5% CO_2_. The media change was every three days for 28 days. The chondrogenic pellet was harvested, embedded in paraffin blocks, sectioned into 5μm tissue sections on glass slides, and stained for cartilage-specific proteoglycan assessment using Alcian blue 8GX stain. (4)

*In vitro adipogenic differentiation*: cells were cultured in regular BMMSC medium until confluence, then switched to adipogenic induction medium for two weeks (low glucose DMEM, 10% FBS, 1% P/S, 1% glutamine, 1μM dexamethasone, 200 µM indomethacin, 0.5 mM 3-isobutyl-1-methyl-xanthine, and 58 µg/mL insulin; all from Sigma-Aldrich, USA). The media was replaced with a differentiation medium containing 10 µg/mL insulin that was refreshed every two days for another week. After three weeks, the cells were fixed with 4% paraformaldehyde (PFA) for 15 min and stained with Oil Red O (Sigma-Aldrich, USA) for lipid accumulation visualization by a phase-contrast inverted microscope. (5)

**2.3** **Immunophenotyping of BMMSCs**

BMMSCs were characterized using fluorescent-labeled monoclonal antibodies. Cells at P3 were trypsinized with 0.25% trypsin-EDTA solution, washed with PBS, and incubated at room temperature in the dark for 30 min with BMMSC marker antibody panel including anti-CD105 primary unconjugated antibody (1 mg/mL, Abcam, UK), anti CD73 primary unconjugated antibody (100 µg/mL, Abcam, UK), anti-CD11b-Phycoerythrin (PE) conjugated antibody (0.2 µg/mL, Abcam, UK), anti-CD45-PE conjugated antibody (200 µg/mL, Abcam, UK), anti-CD90 allophycocyanin-conjugated antibody (Anti-Thy1.1; 100 µg/mL, Abcam, UK) and anti-CD 44 phycoerythrin-(PE-) conjugated antibody 100 µg/mL (Abcam, UK), and then cells centrifuged at 1800 rpm for 10 min and washed with flow cytometry washing buffer. Secondary antibodies, including Goat anti-rabbit IgG-Alexa Fluor 488, and Goat anti-mouse IgG-Alexa Fluor 555 (1:1000, Life Technology, USA), were added for unconjugated antibodies and the tubes were incubated at 4°C for 45 min in the dark. Cells were washed with PBS and resuspended in a 500 µL FACS buffer. The fluorescence of the viable cells was analyzed using BD, FACS caliber flow cytometer operated with Cell Quest software (Becton Dickinson, USA). (6)

**3.Isolation and characterization of small extracellular vesicles (sEVs)**

At P3, the BMMSCs culture media was replaced with serum-free media for 48 h. The conditioned media was collected for sEVs isolation by differential centrifugation. To prepare a concentrated conditioned medium, the conditioned serum-free media was first spun at 300 ×g at room temperature for 5 min to remove any cellular debris. The supernatant was then subjected to high-speed centrifugation at 16,500 ×g at 4°C for 40 min, followed by filtration through a 0.2 µm filter to remove larger microvesicles. Finally, ultracentrifugation of the supernatant (Beckman Coulter, Optima XE Ultracentrifuge with a fixed angle rotor) was pursued at 120,000 ×g at 4°C for 70 min. After discarding the supernatant, the pellet was resuspended in PBS and stored at -80°C for further downstream analysis. (7)

**4.Characterization of Small extracellular vesicles (sEVs)**

**4.1** **Dynamic light scattering**

A diluted sample of sEVs was sonicated to prevent aggregate formations, then placed in the zetasizer sampling tube for size measurement using Malvern Panalytical Software, UK. The measurements were conducted in triplicates under constant equipment settings.(8)

**4.2** **Transmission electron microscopy(TEM)**

The sEVs pellet was dissolved in PBS, loaded to copper grids, and stained with 1% (w/v) phosphotungstic acid (PTA). Samples were examined by TEM with an accelerating voltage of 120 kV (JEM-1400 series 120 kV Transmission Electron Microscope, USA). (9)

**4.3** **Small extracellular vesicles (sEVs)**

**4.4**  **Protein quantification**

The concentration of sEV proteins was determined using the bicinchoninic acid assay (BCA) kit (Sigma-Aldrich, USA). (10)

**4.5** **Immunophenotyping of Small extracellular vesicles (sEVs)**

The surface tetraspanin proteins of the isolated sEVs were further analyzed following a modified protocol using magnetic beads from an SEVs Isolation Kit Pan (Miltenyi 130-117-039) incubated with the sEVs. Briefly, 50 µL of Exosome Isolation MicroBeads was added to the exosome sample, vortexed, and incubated for one hour at room temperature. Following incubation, exosome-bound beads were further washed in PBS/1% BSA, blocked with 10% BSA, and stained with anti-CD9 (CD9 (C-4):sc-13118) conjugated with AlexaFlour^@^488, anti-CD63 antibody (Santa Cruz Biotechnology, MX-49.129.5:sc-5275) conjugated with AlexaFlour^@^647, and anti-CD81 antibody conjugated with AlexaFlour^@^546 1:200:sc-7637), all in 1 µg concentration. MitoTracker, a mitochondrial fluorescent dye, (life technologies cat# M7512, USA) was used as a negative marker. SEVs were incubated with MitoTracker probes, which passively diffuse across their membrane and accumulate in active mitochondria if present. The stained samples were 1h-incubated at room temperature, then washed and resuspended in FACS buffer for further analysis using BD FACSCalibur. (11,12)

**5.MiRNA-21-5p expression**

The expression of miRNA-21-5p was assessed by quantitative reverse transcriptase-polymerase chain reaction (qRT-PCR) in characterized CSCs and different MSCs isolated from available characterized MSCs cultured at CERRMA labs, including BMMSCs, amniotic fluid (AmSCs), and adipose tissue (ATSCs) to select the optimum source with the highest expression for exosome isolation. Briefly, total RNA including miRNAs was extracted from different cell types using miRNeasy kit (Qiagen, Hilden, Germany) according to the manufacturer’s recommendations. The concentration and purity of RNA (OD260/OD280) were determined by a UV-Vis nanodrop spectrophotometer. Single-stranded cDNA was synthesized using miRNA-21 specific primers from TaqMan MicroRNA Assays (Sequence: CAACAGCAGUCGAUGGGCUGUC, assay ID: 002493) using the TaqMan^®^ Reverse Transcription Kit for miRNA #4366596 on a GeneAmp PCR thermal cycler (System 9700 N8050200) with the following settings: 15.0 µL reaction, 16°C for 30 min, 42°C for 30 min 85°C for 5 min and 4°C on hold, cDNA was stored at -20 until qPCR experiments. miRNA-21 was assayed using TaqMan MicroRNA Assays #4427975 using Bio-Rad CFX96™ Real-Time PCR detection system under the following settings: 20 µL reaction, 50°C for 2 min, 95°C for 10 min, 95°C for 15 sec and 60°C for 60 sec using TaqMan^®^ miRNA-21 Assay, TaqMan^®^ 2× Universal PCR Master Mix^®^, UNG #4440042 and U6 snRNA as an endogenous control (Sequence: GTGCTCGCTTCGGCAGCACATATACTAAAATTGGAACGATACAGAGAAGATTAGCATGGCCCCTGCGCAAGGATGACACGCAAATTCGTGAAGCGTTCCATATTTT, assay ID: 001973). All reagents were obtained from Applied Biosystems, USA. miRNA-21 level calculation was done using the Livak (2^–ΔΔCT^) method. (7,13)

**6. Isolation and characterization of CSCs**

Six BALB/c mice (6 weeks old and 25-30 g) were anesthetized with an intraperitoneal injection of 1000 IU/Kg heparin sulfate. Heart exposure was through a vertical cut along the thoracic cage, and the ascending aorta was cannulated with a fine-bore needle for the retrograde perfusion of Basic buffer at a rate of 10 mL/min for 5 min using a perfusion pump (138762, Fisher Scientific) to wash the whole blood. The basic buffer comprises MEM (Joklik modification) containing sodium bicarbonate (2 g/L), HEPES (0.7 mg/mL), taurine (1.25 mg/mL), and glutamine (0.3 mg/mL). The apex of the heart was pierced to ensure drainage of any pooled blood. Perfusion was then continued with the digestive collagenase solution (collagenase type II 250U/mL; Roche, cat#11088866001) in the basic buffer for 10-15 min until the heart enlarged slightly and the tissue became pale and flaccid. Then, heart digestion was blocked using BSA solution perfusion (5 mg/mL BSA in Basic buffer) for another 5 min. Further tissue dissociation was pursued mechanically with separation of the cell fractions by centrifugation (300 ×g for 1 min at room temperature), followed by filtering the supernatant using a 40 µm cell strainer to obtain cell fraction with further centrifugation (300 ×g for 7 min). (14)

For CSCs purification from cardiac mast cells, magnetic-activated cell sorting technology was performed to remove CD45^+^ cells using the EasySepTM Mouse CD45 positive selection kit (Stem cell Technology cat# 18757) following the manufacturer protocol. Then, the negatively selected purified CSCs were cultured on 2%gelatin coated plates (Sigma- Aldrich, cat.no G1890) in an equal mixture of DMEM-F12-Ham’s (supplemented with 1% of both insulin-transferrin-selenium, P/S-fungizone, and 0.1% gentamicin) and neurobasal medium (supplemented with 1% l-glutamine, 2% B27 supplement, and 1% N2 supplement from Invitrogen, cat# 17502-048). Then, the following factors were added to the media mixture, including 10% FBS, epidermal growth factor (20 ng/mL, Peprotech, cat# 100-15), basal fibroblast growth factor (10 ng/mL, Peprotech, cat# 100-18B), and leukemic inhibitory factor (10 ng/mL, Millipore, cat# LIF2010). (14)

**7. Characterization of CSCs**

**7.1** **Immunofluorescent staining**

Once confluent, CSCs were washed with PBS and fixed with 4% PFA for 10 min at 37°C. The cells were then permeabilized using 0.1% Triton X-100 for 15 mins at room temperature and blocked for 1 h using 2% BSA. CSCs immunofluorescent staining was performed using GATA4 (Abcam ab227512) primary rabbit monoclonal antibodies in a dilution of 1:100. CSCs were incubated for 45 min at room temperature with Goat anti-rabbit secondary antibodies (Abcam ab150077) in 1:200 dilutions and Hoechst-counterstained in a dilution 1:1000. Immunostained cells were visualized using a confocal microscope (Leica TCS SP5, Germany). (15)

**7.2** **CSCs surface characterization**

At P3, cell surface staining was carried out using anti-CD45-PE conjugated antibody (200 µg/mL, Abcam, UK), anti-CD90-fluorescein isothiocyanate (FITC; 30 µg/mL, Miltenyi, Biotec, Germany), anti-CD117-PE conjugated antibody (50 µg/mL, Stem cell, UK) anti-CD105 (1 mg/mL, Abcam, UK), anti-CD73 (1 mg/mL, BD Bioscience, Becton, USA), anti-CD Sca1 (0.3 mg/mL, Abcam, UK), and anti-OCT4 (0.672 mg/mL, Abcam, UK), followed by secondary antibody staining with anti-mouse IgG conjugated to Alexa 555 (1:1000, Life Technology, Eugene, USA). The fluorescence of the viable cells was analyzed using BD, FACS Calibur flow cytometer operated with Cell Quest software (Becton Dickinson, USA). (16)

**8. Induction of ischemia-reperfusion injury**

To mimic the cardiac ischemia model, in vitro cultured primary CSCs were subjected to oxygen-glucose deprivation (OGD), followed by reoxygenation on three independent samples (n=3) for each experiment.

Oxygen deprivation was induced using a hypoxic chamber (stem cell technologies, 27310) to simulate hypoxia by creating a self-contained, sealed, and low-oxygen environment. Per manufacturer protocol, the hypoxic chamber was purged with a gas mixture of 1% O2 and 5% CO2 balanced with nitrogen at a rate of 20 L/min for 5 min.

At passage 3, CSCs were seeded in 6-well plates with a cell density of 3 × 105 cells per well in complete growth media. After 24 h, cells were washed twice with PBS and prepared for glucose deprivation by adding DMEM without glucose and pyruvate. Plates were then placed in the hypoxic chamber and sealed in the incubator for 24 h for the induction of hypoxia. (16)

**9. Assessment**

**9.1. A** **Hypoxia-induced factor-1α flow cytometry assessment**

After 24 h for the OGD group and 72 h for OGD/R and ExT groups, cells were fixed with 4% PFA for 10 min at 37°C, permeabilized using 0.1% Triton X-100 for 15 mins at room temperature, then blocked for 1 h using 2% BSA and finally incubated primary monoclonal anti-HIF-1α ab [H1alpha67] (ab1). Suspended cells were then labeled with anti-mouse Alexa-Fluor^®^ 555 dye for analysis using a BD FACSCalibur flow cytometer. While cells grown on chamber slides were labeled Goat anti-mouse-PE (life technology A21422) in 1:200 dilutions for 45 min at room temperature, and DAPI was added as a counterstain with the secondary antibodies in a dilution 1:1000. Visualization of the immunofluorescent stained cells was through the confocal microscope (Leica TCS SP5, Germany). HIF-1α gene expression was also assessed using qRT-PCR, as described before.

9.1.B *Hif-*1α relative gene expression using reverse transcription quantitative polymerase chain reaction (RTqPCR)

CSCs were washed twice with ice-cold PBS, lysed by 500 µL Qiazole and frozen at −80 °C until RNA isolation. Total RNA was extracted by a spin protocol (Qiagen RNeasy Mini Kit #74104). RNA concentrations and quality (260/280 ratio) were determined on a Thermo Scientiﬁc ND2000 Nanodrop Spectrophotometer and stored at −80 °C. Total RNA (25 ng/μL) was reverse transcribed using the high-capacity cDNA reverse transcription kit (Life Technologies #4374966) on an Applied Biosystems GeneAmp PCR System 9700 N8050200 thermal cycler with the following settings: 25 °C for 10 min, 37 °C for 120 min, 85 °C for 5 s, and 4 °C on hold. cDNA was stored at -20 until qPCR experiments. Gene-speciﬁc regions were ampliﬁed from cDNA with assay primers (100 nM each; Invitrogen #10629186) and Maxima SYBR Green/ROX kit (Thermo Scientific #K0251) on a Bio-Rad CFX96™ Real-Time PCR Detection System with the following settings: 20 μL reaction, 95°C for 10 min, followed by 40 cycles; 95°C for 15s, 60°C for 30s and 72°C for 30 s. Gene expression analysis was performed using the relative quantiﬁcation (ΔΔCt) method. Results are presented as fold change relative to *Gapdh* (2^−ΔΔCt^). Primer sequences are listed in Table S1.

Table S1. Primer sequences used in quantitative real-time PCR (qRT-PCR).

| **Primer sequences** | **GENE** |
| --- | --- |
| **Fwd:** 5`- CCTGCACTGAATCAAGAGGTTGC-3`  **Rev:** 5`- CCATCAGAAGGACTTGCTGGCT-3` | ***Hif1α*** |
| **Fwd:** 5`- CTGCGACTTCAACAGCAACT-3`  **Rev:** 5`- GAGTTGGGATAGGGCCTCTC-3` | ***Gadph*** |

**9.2** **Cell proliferation assays**

**9.2.1** **Cell cycle assay**

Cell cycle assay was performed by PI/RNase reagent (cat# 4087S, Cell Signaling, life technology, USA). After centrifugation of the cells at 1800 rpm for 5 min, the pellet was washed with 1 mL PBS (1 mL of PBS per 10^6^ cells). After re-centrifugation, the cell pellet was resuspended in 1 mL ice-cold 95% methanol for 60 min. Methanol-fixed cells were centrifuged at 1800 rpm for 5 min at room temperature, and the pellet was resuspended in 6 mL PBS to remove excess methanol. The cells were recentrifuged (1800 rpm for 5 min at room temperature), and the cell pellet was resuspended in 200 µL of PI/RNase reagent for 15 min incubation at room temperature in the dark. The stained cell suspension was ready to be analyzed on BD FACSCalibur flow cytometry.

**9.2.2** **Proliferative maker Ki67**

Cells were fixed with 4% PFA for 10 min at 37°C, permeabilized using 0.1% Triton X-100 for 15 mins at room temperature, then blocked for 1 h using 2% BSA and finally incubated with the Alexa Flour 488-conjugated anti-rabbit Ki67 antibody (IgG, Cell Signaling Technology, USA, cat# 11882S), assessment and data analysis were done using BD FACSCalibur flow cytometry.

**9.2.3** **EdU DNA staining**

EdU staining was carried out with Click-iT™ EdU Alexa Fluor™ 594 Flow Cytometry Assay Kit (cat# C10420, Life Technologies, 29851 Willow Creek Road, Eugene, USA) according to the manufacturer protocol. EdU was added to the culture medium at a final concentration of 10 µM for 1–2 h and then mixed well. Cells were harvested in appropriate tubes and washed once with 3 mL of 1% BSA in PBS by centrifugation. Then, the cell pellet was incubated with 100 µL of Click-iT^®^ fixative for 15 min at room temperature in the dark. Cells were washed with 3 mL of 1% BSA in PBS with supernatant discard. Cells were incubated with 100 µL of 1X Click-iT^®^ saponin-based permeabilization and wash reagent for 15 min at room temperature. Then, the EdU reaction cocktail, prepared according to manufacturer protocol, was incubated with cells for 30 min. Cells were washed with 3 mL of 1% BSA in PBS, followed by another wash with 3 mL of 1X Click-iT^®^ saponin-based permeabilization wash reagent. Flow cytometry was used for analyzing EdU staining.

**10.** **Apoptosis**

Cell pellets from all groups were washed in PBS and resuspended in 100 µL Annexin V binding buffer (1x). Then, Annexin V Alexa Fluor 488 (5 µL, BD Biosciences Pharmingen TM, USA) and PI (5 µL) were added for 15 min incubation in the dark at room temperature. Annexin/PI-stained cells were evaluated according to the previously described method on a BD FACS Calibur instrument (Becton Dickinson, USA) fitted with a 488 nm argon laser. A minimum of 10,000 cells per sample were acquired and analyzed using Cell Quest Pro software.

**11.** **Oxidative stress profile**

**11.1** **Malondialdehyde (MDA)**

After adding 0.5 ml of the culture media to 1 ml of TCA, the mixture was centrifuged for 10 minutes at 3000 r.p.m. After adding 0.5 ml of TBA (0.7%) to 1 ml of supernatant, the mixture was incubated for 45 minutes in a boiling water bath.

0.5 ml of distilled water was used for the blank experiment. A spectrophotometer was then used to measure the colour at 532 nm against the blank reagent. The following formula was used to calculate the MDA concentration: nmol/ml MDA concentration = At / 0.15. The extension coefficient was 0.156.(17)

**11.2** **Total Antioxidant Capacity** (**TAC)**

The total antioxidant capacity Colorimetric Method kit (Bio Diagnostic and Research Reagents® CAT. No. TA 25 13) was used to measure the total antioxidant capacity in cell culture media. The antioxidants in the sample reacted with a certain quantity of exogenously supplied hydrogen peroxide (H2O2) to determine the antioxidative capacity. A specific amount of the supplied hydrogen peroxide is eliminated by the antioxidants in the sample. Through an enzymatic reaction that involves the conversion of 3,5, dichloro-2-hydroxy benzenesulfonate to a colored product, the residual H2O2 was measured colorimetrically. (18)

**11.3** **Oxidative stress index (MDA/TAC index)**

Indirect measurement of oxidative stress status was calculated as a ratio of the lipid peroxidation marker malondialdehyde (MDA, measured in nmol/mL) to the total antioxidant capacity (TAC, measured in nmol/mL), collectively known as MDA/TAC oxidative stress index.

**12.** **PTEN /Akt/ pAkt/ HIF-1α pathway**

For evaluation of the downstream PTEN/Akt/pAkt/HIF-1α pathway targeted by miRNA-21, protein expression was assessed by western blot. Total proteins were extracted by lysing CSCs in RIPA lysis buffer (cat# 9806s) and Protease/Phosphatase Inhibitor Cocktail (cat# 5872). Equivalent amounts of protein (30 μg), as determined by standard Bradford assay, were loaded and separated by sodium dodecyl sulfate (SDS) polyacrylamide gel electrophoresis (SDS-PAGE) and transferred to Nitrocellulose membranes (cat# 12369P2) using a transfer apparatus. After incubation with 5% non-fat milk in TBST (10mM Tris pH 8.0, 150mM NaCl, 0.5% Tween 20) for 60 minutes, membranes were washed twice with TBST and incubated overnight at 4°C with a 1:1000 dilution of primary antibodies against PTEN, AKT, Phospho-Akt, and β-actin using PTEN (138G6) Rabbit mAb (cat# 9559S), Akt (pan) (C67E7) Rabbit mAb (cat# 4691S), Phospho-Akt (Ser473) (D9E) XP® Rabbit mAb (cat# 4060S) and β-Actin (13E5) Rabbit mAb (cat# 4970s), respectively. Then, membranes were incubated for 1 h at room temperature with a 1:2000 dilution of the horseradish peroxidase-conjugated Anti-rabbit IgG antibody (cat# 7074s). All antibodies were obtained from Cell Signaling Technology, Inc. Blots were washed twice with TBST and developed with the chromogenic western blot detection using Thermo Scientific™ Step™ Ultra TMB-Blotting Solution (cat# 37574). The density of the bands was assessed using BIO RAD Gel DocTM XR with Image Lab TM software version 5.1 for band imaging and densitometry.

**References**

1. Ohkawa H, Ohishi N, Yagi K. Assay for lipid peroxides in animal tissues by thiobarbituric acid reaction. Analytical biochemistry. 1979;95(2):351–8.

2. Koracevic D, Koracevic G, Djordjevic V, Andrejevic S, Cosic V. Method for the measurement of antioxidant activity in human fluids. Journal of clinical pathology. 2001;54(5):356–61.

1. Khalifa YH, Mourad GM, Stephanos WM, Omar SA, Mehanna RA. Bone Marrow-Derived Mesenchymal Stem Cell Potential Regression of Dysplasia Associating Experimental Liver Fibrosis in Albino Rats. Biomed Res Int. 2019;5376165.

2. Franken NA, Rodermond HM, Stap J, Haveman J, Bree C. Clonogenic assay of cells in vitro. Nat Protoc. 2006;1(5):2315–9.

3. Hu Y, Lou B, Wu X, Wu R, Wang H, Gao L, et al. Comparative Study on In Vitro Culture of Mouse Bone Marrow Mesenchymal Stem Cells. Stem Cells Int. 2018;6704583.

4. Abolgheit S, Abdelkader S, Aboushelib M, Omar E, Mehanna R. Bone marrow-derived mesenchymal stem cells and extracellular vesicles enriched collagen chitosan scaffold in skin wound healing (a rat model. J Biomater Appl. 2021;36(1):128–39.

5. Li X, Zhang Y, Qi G. Evaluation of isolation methods and culture conditions for rat bone marrow mesenchymal stem cells. Cytotechnology. 2013;65(3):323–34.

6. Martins AA, Paiva A, Morgado JM, Gomes A, Pais ML. Quantification and immunophenotypic characterization of bone marrow and umbilical cord blood mesenchymal stem cells by multicolor flow cytometry. Transplant Proc. 2009;41(3):943–6.

7. Thabet E, Yusuf A, Abdelmonsif DA, Nabil I, Mourad G, Mehanna RA. Extracellular vesicles miRNA-21: a potential therapeutic tool in premature ovarian dysfunction. Vol. 26. Mol Hum Reprod; 2020. 906–919 p.

8. Chiang C yi, Chen C. Toward characterizing extracellular vesicles at a single-particle level. Journal of biomedical science. 2019;26:1–10.

9. Xu R, Greening DW, Zhu HJ, Takahashi N, Simpson RJ. Extracellular vesicle isolation and characterization: toward clinical application. J Clin Invest. 2016;126(4):1152–62.

10. Matsuura K, Nagai T, Nishigaki N, Oyama T, Nishi J, Wada H, et al. Adult cardiac Sca-1-positive cells differentiate into beating cardiomyocytes. J Biol Chem. 2004;279(12):11384–91.

11. Sedik AS, Kawana KY, Koura AS, Mehanna RA. Biological effect of bone marrow mesenchymal stem cell- derived extracellular vesicles on the structure of alveolar bone in rats with glucocorticoid-induced osteoporosis. BMC Musculoskelet Disord. 2023;24(1):205.

12. Ashour AA, El-Kamel AH, Mehanna RA, Mourad G, Heikal LA. Luteolin-loaded exosomes derived from bone marrow mesenchymal stem cells: a promising therapy for liver fibrosis. Drug Deliv. 2022;29(1):3270–80.

13. Livak KJ, Schmittgen TD. Analysis of relative gene expression data using real-time quantitative PCR and the 2(-Delta Delta C(T)) Method. Methods. 2001;25 402-408.

14. Smith AJ, Lewis FC, Aquila I, Waring CD, Nocera A, Agosti V, et al. Isolation and characterization of resident endogenous c-Kit+ cardiac stem cells from the adult mouse and rat heart. Nat Protoc. 2014;9(7):1662–81.

15. Belostotskaya G, Nevorotin A, Galagudza M. Identification of cardiac stem cells within mature cardiac myocytes. Cell Cycle. 2015;14(19):3155–62.

16. Chen T, Vunjak-Novakovic G. In vitro Models of Ischemia-Reperfusion Injury. Regen Eng Transl Med. 2018;4(3):142–53.

17. Ohkawa H, Ohishi N, Yagi K. Assay for lipid peroxides in animal tissues by thiobarbituric acid reaction. Analytical biochemistry. 1979;95(2):351–8.

18. Koracevic D, Koracevic G, Djordjevic V, Andrejevic S, Cosic V. Method for the measurement of antioxidant activity in human fluids. Journal of clinical pathology. 2001;54(5):356–61.

19. Paglia DE, Valentine WN. Studies on the quantitative and qualitative characterization of erythrocyte glutathione peroxidase. The Journal of laboratory and clinical medicine. 1967;70(1):158–69.

**Supplementary figures**

**
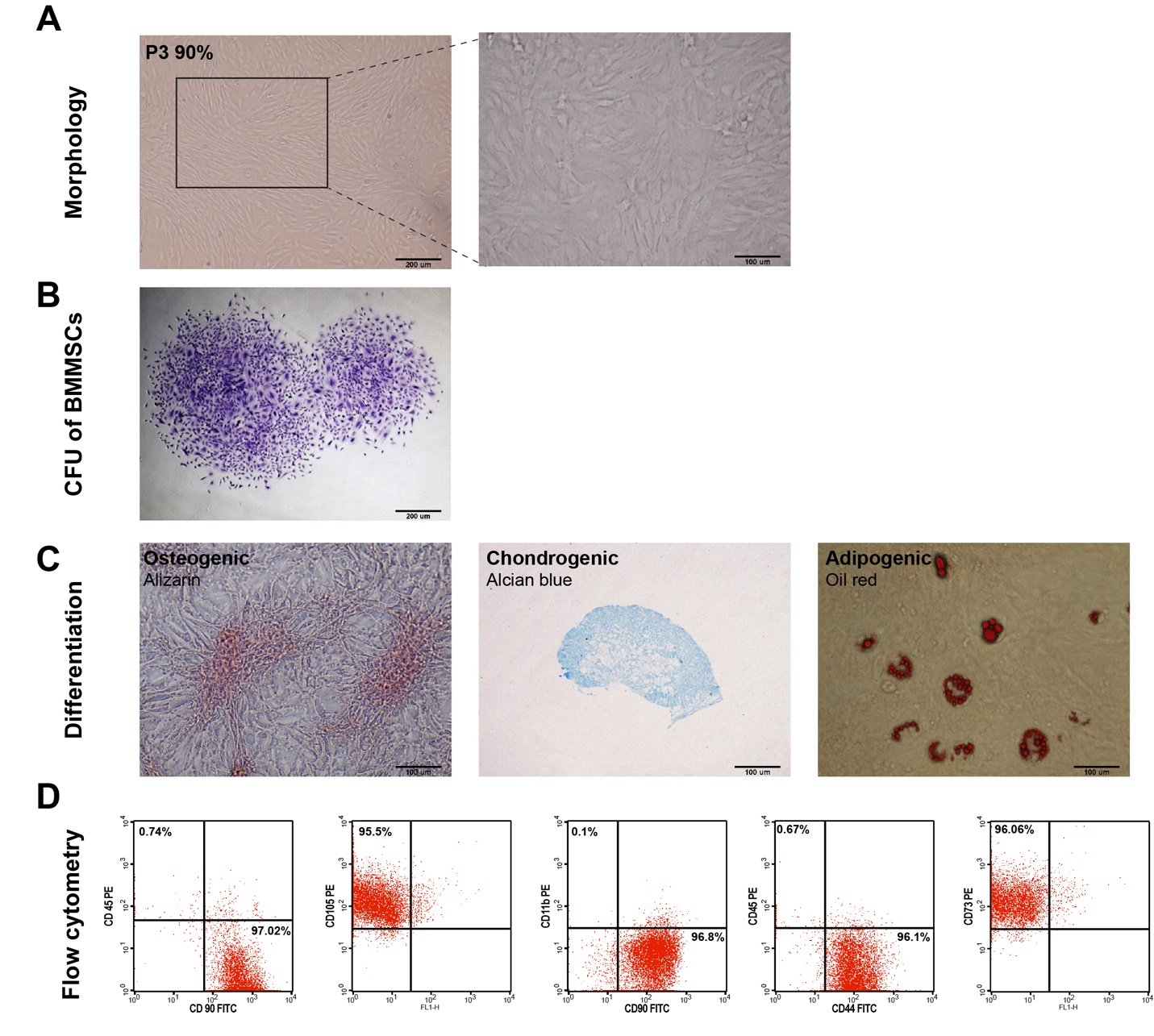
Supplementary Figure 1. Characterization of BMMSCs. (A)** Phase contrast microscopic images of isolated BMMSCs at passage 3 (P3) and 90% confluence (×100 with scale bar 200 µm and its high power ×200 with scale bar 100 µm). **(B)** A crystal violet-stained contrast phase microscopic image shows the colony forming unit (CFU) potentiality of the isolated BMMSCs (×100 = 200 µm). **(C)** Phase contrast microscopic images (×200 with scale bar 100 µm) of positive BMMSCs differentiation into bone-forming cells (Alizarin-stained), cell pellet containing cartilage-forming cells (Alcian blue-stained cell pellet), and adipose-forming cells (oil red-stained). **(D)** Flow cytometry assay displays the percentage expression of BMMSCS markers (CD45, CD90, CD105, CD11b, CD44, CD73).


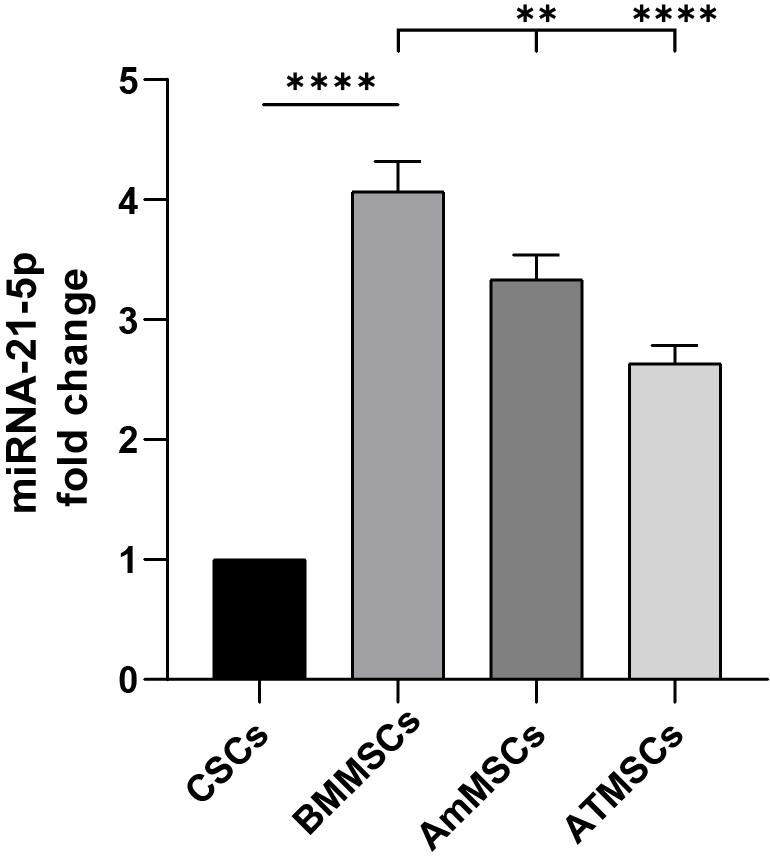


**Supplementary Figure 2.** The relative miRNA-21 expression (fold change) in CSCs compared to different MSCs; BMMSCs, AmMSCs, and ATMSCS assessed by RT-qPCR testing. The results are expressed as mean ± SD of triplicates from 3 independent experiments, where **p < 0.01 and ****p < 0.0001.


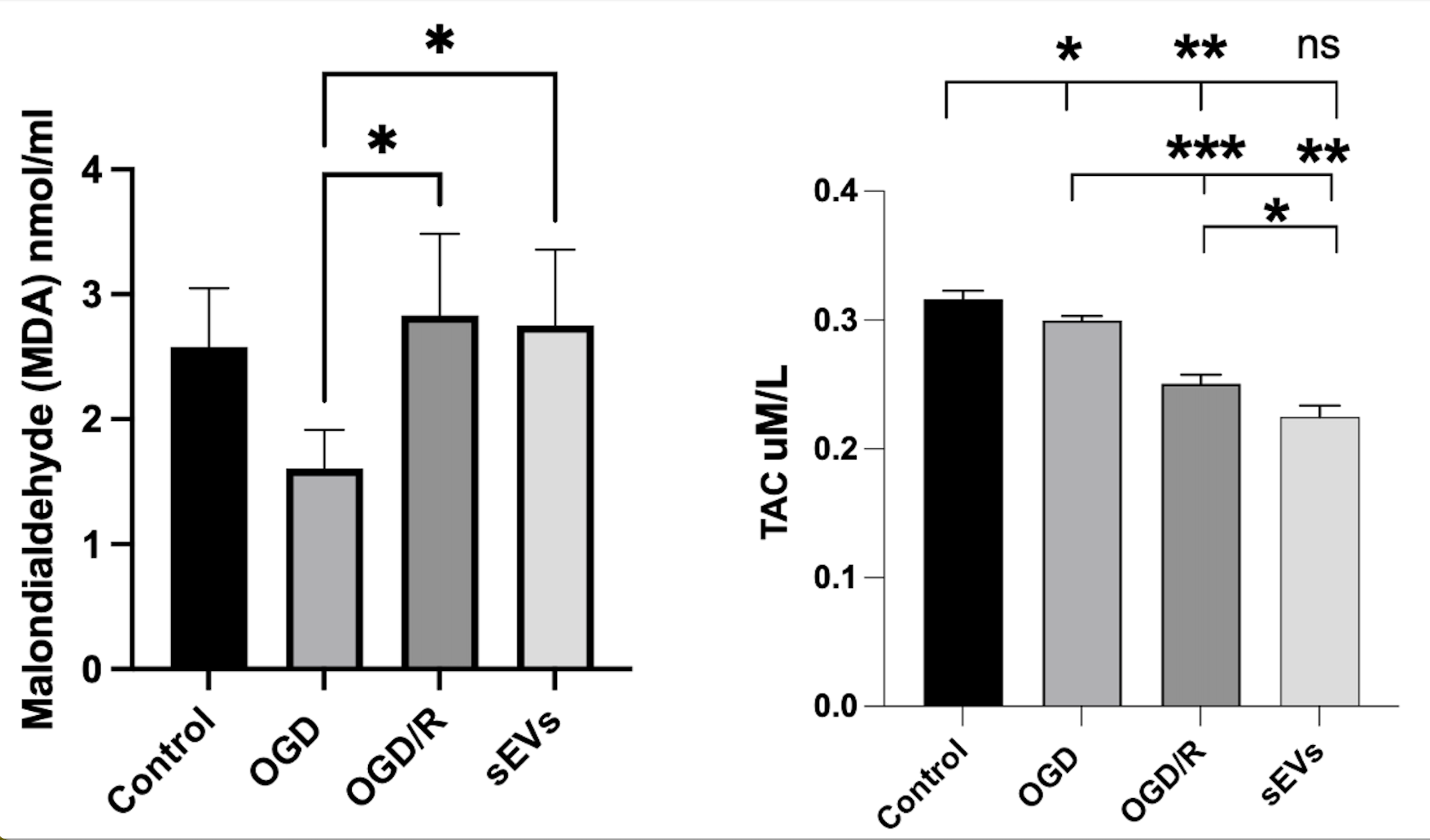


**Supplementary Figure 3:** Mean Malondialdehyde (MDA)(nmol/ml) and Total Antioxidant Capacity (TAC) uM/L) in cell culture media among CSC/Sca1+ groups. Data in each bar chart are representative of mean ± SD of 3 independent experiments performed in triplicates each (each replica consists of media from 25×10^4^ CSCs seeded into 3 wells of 6-well plates), where one-way ANOVA followed by Tukey’s multiple comparisons test reveals **p* < 0.05, ***p* < 0.01, and ****p* < 0.001, while ns means non-significance of *p* > 0.05.
